# Rapid access to phospholipid analogs using thiol-yne chemistry†
†Electronic supplementary information (ESI) available: Details of experimental procedures, lipid characterization data (NMR, HRMS, steady-state fluorescence anisotropy), HPLC analysis, fluorescence microscopy images, liposome shrinkage data in the form of AVI files. See DOI: 10.1039/c5sc00653h


**DOI:** 10.1039/c5sc00653h

**Published:** 2015-05-19

**Authors:** Cun Yu Zhou, Haoxing Wu, Neal Krishna Devaraj

**Affiliations:** a Department of Chemistry and Biochemistry , University of California , San Diego , La Jolla , California 92093 , USA . Email: ndevaraj@ucsd.edu

## Abstract

Phospholipids and glycolipids constitute an essential part of biological membranes, and are of tremendous fundamental and practical interest. Unfortunately, the preparation of functional phospholipids, or synthetic analogs, is often synthetically challenging. Here we utilize thiol-yne click chemistry methodology to gain access to phospho- and glycolipid analogs. Alkynyl hydrophilic head groups readily photoreact with numerous thiol modified lipid tails to yield the appropriate dithioether phospho- or glycolipids. The resulting structures closely resemble the structure and function of native diacylglycerolipids. Dithioether phosphatidylcholines (PCs) are suitable for forming giant unilamellar vesicles (GUV), which can be used as vessels for cell-free expression systems. The unnatural thioether linkages render the lipids resistant to phospholipase A2 hydrolysis. We utilize the improved stability of these lipids to control the shrinkage of GUVs composed of a mixture of 1,2-dioleoyl-*sn*-glycero-3-phosphocholine (DOPC) and dioleyl-dithioether PC, concentrating encapsulated nanoparticles. We imagine that these readily accessible lipids could find a number of applications as natural lipid substitutes.

## Abstract 2

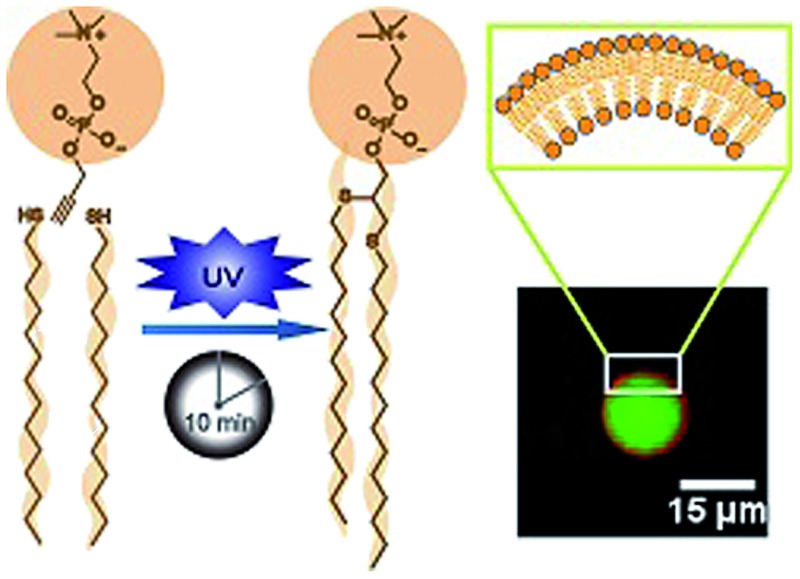
Membrane-forming phospholipids analogs can be rapidly formed through use of thiol-yne click chemistry.

## Introduction

Phospholipids represent the most commonly utilized building block of cell membranes in living organisms. Phospholipid bilayers provide enclosed environments vital for cells to maintain cellular contents and to facilitate cellular biochemistry.^1^ Phospholipids and their derivatives have been extensively utilized for the formation of liposomes for drug delivery^2,3^ and for the construction of artificial cells.^4–8^ There is also a high demand for using phospholipids as therapeutic agents, such as pulmonary surfactant.^9^ In spite of their many essential biological functions and material and pharmaceutical applications, straightforward methods to synthesize phospholipids in high yield are limited.^10^ Phospholipid synthetic methods often suffer from lengthy syntheses, arduous work-up and low yield.^11,12^ A click chemistry approach to phospholipid synthesis could offer an excellent solution to these problems by providing many synthetic advantages, including fast reaction rate, high chemoselectivity, and good yields.^5^ Previously we demonstrated the use of copper catalyzed alkyne-azide cycloadditions to *in situ* generate phospholipids that would consequentially form artificial membranes.^5^ In this study, we report the use of thiol-yne click chemistry to rapidly and efficiently synthesize dithioether phospho- and glycolipids that mimic key structural features of natural lipids.

The discovery of radical initiated thiol-yne and thiol-ene photo click chemistry introduced a powerful synthetic tool suitable for a wide range of applications,^13–16^ including building polymers^17–21^ and polymeric networks,^22–27^ microcontact printing,^28,29^ surface engineering,^20,25,30–34^ selective glycosylation and glycoconjugation of biomolecules^23–25^ and drug delivery.^24,35–42^ Recent elegant studies have shown the formation of amphiphiles using thiol-yne chemistry for siRNA and gene delivery.^42,43^ However, the creation of membrane forming phospholipids was not examined. Thiol-yne click chemistry offers numerous advantages for the synthesis of dithioether phospho- and glycolipids. It requires only one step to assemble three components to access the final lipid product, proceeds with fast reaction rates, produces minor side products, and shows excellent chemoselectivity and functional group tolerance enabling the use of unprotected glycans and olefins. Changing the alkyne and thiol precursors allows access to numerous lipid analogs. The resulting dithioether phosphatidylcholines (PCs) offer a biocompatible alternative to natural PCs to form liposomes capable of hosting biochemical reactions. The resistance of dithioether PCs to hydrolysis by enzymes, such as phospholipases, enables shrinkage of liposomes and the concentration of encapsulated nanomaterials. We imagine that dithioether lipids could have numerous applications such as mimicking the cell membrane for micro-bioreactors, containing and concentrating large biomolecules and nanoparticles for drug delivery applications, and developing novel lung surfactants.^44–46^

## Experimental

### General methods

Prop-2-ynyle phosphatidylcholine was synthesized according to a previously published procedure.^35^ All reagents were purchased from Sigma-Aldrich and used without further purification unless otherwise noted. All diacylglycerol phospholipids were purchased from Avanti Polar Lipids, Inc. (Alabaster, AL). pBEST-OR2-OR1-Pr-UTR1-deGFP-T500 was a gift from Vincent Noireaux (Addgene plasmid # 40019). Chinese hamster ovary cells were received from Professor Kamil Godula (University of California, San Diego). Full synthetic details for **2–8** are provided in the ESI.† The products were purified by silica column chromatography by using 40–63 μm silica gel. All mixtures of solvents are given in v/v ratio. ^1^H and ^13^C NMR spectroscopy was performed on a Jeol NMR at 500 (^1^H) or 126 (^13^C) MHz. All ^13^C NMR spectra were proton decoupled. Fluorescence steady-state anisotropy was measured with a Perkin Elmer LS55 Luminescence Spectrometer (Waltham, MA). Fluorescence microscopy images were acquired on an Axio Observer D1 inverted microscope (Carl Zeiss Microscopy GmbH, Germany) with a 20× and 40×, 0.6 NA air immersion objective and ORCA-ER camera (Hamamatsu, Japan) using Micro-manager 7.4.12 software. Fluorophores were excited with an HXP 120 metal halide arc lamp (Carl Zeiss Microscopy GmbH, Germany). HPLC traces were monitored using an Agilent 1260 Infinity Series HPLC with an Agilent Zorbax eclipse plus C8 column, a 380 Varian-Agilent ELSD and a 6100 Agilent Quadrupole MS (Santa Clara, CA).

### General procedure for synthesis of dithioether PC using photo-initiated thiol-yne chemsitry

Prop-2-ynyle phosphatidylcholine^47^ (25 mg, 0.11 mmol) and alkylthiol (0.57 mmol, 5 eq.) were sonicated under N_2_ protection for 60 min in 3 mL DMF. The mixture was then transferred to a 3500 μL macro fluorescence cuvette, after which 2,2-dimethoxy-2-phenylacetophenone (DMPA) (2 mg, 0.008 mmol) was added. The reaction was exposed to UV light (354 nm) for 10 min. The solvent was removed and the residue was purified by column chromatography (DCM : MeOH : H_2_O = 5 : 1 : 0.1). Representative characterization data of C12:0 dithioether PC **1**: waxy solid, 65.7 mg, 93% yield. ^1^H NMR (500 MHz, CDCl_3_) *δ* 4.30 (s, 2H), 4.10 (s, 1H), 3.90 (s, 1H), 3.80 (s, 2H), 3.38 (s, 9H), 2.97–2.89 (m, 1H), 2.88–2.82 (m, 1H), 2.80–2.73 (m, 1H), 2.53 (dd, *J* = 15.7, 8.0 Hz, 4H), 1.60–1.46 (m, 4H), 1.34 (s, 4H), 1.24 (s, 32H), 0.86 (t, *J* = 7.0 Hz, 6H). ^13^C NMR (126 MHz, CDCl_3_) *δ* 76.70, 66.53, 59.41, 54.54, 54.54, 54.54, 46.48, 34.61, 33.30, 32.06, 32.06, 31.68, 30.12, 29.96, 29.83, 29.83, 29.80, 29.80, 29.80, 29.80, 29.75, 29.75, 29.51, 29.51, 29.48, 29.48, 29.20, 29.14, 22.82, 22.82, 14.27, 14.27. HRMS [M + Na]^+^ *m*/*z* calcd. for [C_32_H_68_NO_4_PS_2_Na]^+^ 648.4220, found 648.4221.

### Lipid driven formation of dithioether PC

Prop-2-ynyle phosphatidylcholine (4.2 mg, 19 μmol) was dissolved in 210 μL H_2_O containing 35 mM 1,2-dipalmitoyl-*sn*-glycero-3-phosphocholine (DPPC). Dodecanethiol (45 μL, 190 μmol) and 2-hydroxy-4′-(2-hydroxyethoxy)-2-methylpropiophenone (1.5 mg, 6.7 μmol) were well mixed in the solution by sonication under a N_2_ atmosphere. The reaction was exposed to UV light (354 nm) for 75 min. The reaction was analyzed by LCMS-ELSD. HPLC gradient: 0–1 min 50% Phase B in Phase A to 100% Phase B, 1–7 min 100% Phase B, 7–8 min 100% Phase B to 50% Phase B in Phase A (Phase A: H_2_O with 0.1% formic acid, Phase B: MeOH with 0.1% formic acid).

### Anisotropy Measurements

Anisotropy experiments were performed as previously reported.^5,48^ Steady-state anisotropy was measured on a Perkin Elmer LS55 Luminescence Spectrometer with a refrigerated circulating water bath for temperature control. Anisotropy was calculated as a unitless ratio defined as Anisotropy = (*I*_vv_ – (GF × *I*_vh_))/(*I*_vv_ + (2 × GF × *I*_vh_)), where *I*_vv_ is the intensity with polarizers vertical and vertical (excitation and emission) and *I*_vh_ is the intensity with polarizers vertical and horizontal (excitation and emission) at 420 nm (excitation 350 nm). GF is the grating factor that corrects for efficiency differences in the instrument optics defined as GF = *I*_hv_/*I*_hh_, where *I*_hv_ is the intensity with polarizers horizontal and vertical (excitation and emission) and *I*_hh_ is the intensity with polarizers horizontal and horizontal (excitation and emission).^49^ Measurements were taken every 3 °C except when close to the phase transition temperature (*T*_C_) of the lipid, in which case they were taken every 1 or 2 °C. 1,6-Diphenyl-1,3,5-hexatriene (DPH) anisotropy as a function of temperature was used to measure the phase transition of the lipid chains in dithioether PC vesicles. The sudden change in the slope of the anisotropy indicates a transition from a gel to liquid-crystalline phase.^50^

### General procedure for synthesis of giant unilamellar vesicles (GUVs)

The GUV formation procedure was adopted and modified from a previously published method.^51^ In a 2 mL vial, 40 μL of 10 mM lipid solution in chloroform was dried under N_2_ to form a lipid film. 200 μL of light mineral oil was added to the vial and the mixture was sonicated at 60 °C for 1 h until the lipid was fully dissolved in the oil. In a 0.7 mL Eppendorf tube, 10 μL of the upper buffer (100 mM HEPES, 200 mM sucrose, 1 mM 8-hydroxypyrene-1,3,6-trisulfonic acid (HPTS) in H_2_O, pH = 7.4) was added to 100 μL of lipid solution. The mixture was flicked to form an emulsion. The emulsion was gently layered on top of 100 μL of lower buffer (100 mM HEPES, 200 mM glucose in H_2_O, pH = 7.4) in a different tube and the mixture was centrifuged at 10 000 rcf for 5 min. Light mineral oil was removed by vacuum suction and the vesicle solution was obtained. 0.1 μL of 100 μM Texas Red DHPE (Invitrogen, Carlsbad, CA) solution in EtOH was added to 10 μL of vesicle solution prior to microscope observation.

### General procedure for giant unilamellar vesicle shrinking

Mixed 1,2-dioleoyl-*sn*-glycero-3-phosphocholine (DOPC)/C18:1 dithioether PC **5** (molar ratio: 85 : 15) GUVs were prepared and characterized using the aforementioned procedure, with the option of encapsulation of 0.1% red fluorescent sulfate-modified polystyrene nanoparticles (Sigma-Aldrich, St. Louis, MO), or 1 mg mL^–1^ superfolder green fluorescent protein (sfGFP) as a fluorescent indicator. The sfGFP with his-tag was purified by a nickel resin column. For the GFP GUV shrinkage experiment, 10 mM CaCl_2_ and 35 mM KCl were added in all buffers. For GUV shrinkage experiments with fluorescent nanoparticles, 0.1% Tergitol Type NP-40 surfactant was introduced to the buffer containing nanoparticles to avoid aggregation. 0.5 μL of 100 mM Texas Red DHPE or NBD-PE (Invitrogen, Carlsbad, CA) was added to 100 μL vesicle suspension, which was then added to a 96-well plate. GUVs were allowed to settle to the bottom of the plate. 50 μL of 20 μg mL^–1^ bee venom phospholipase A_2_ (PLA_2_) (Sigma-Aldrich, St. Louis, MO) solution was added to the sample to give a final PLA_2_ concentration of 6.67 μg mL^–1^. Time-lapse fluorescence microscopy was used to monitor the process of vesicle shrinkage.

### Cell viability study

Chinese hamster ovary (CHO) cells were plated in a 96 well tissue culture plate in cDMEM medium (10% fetal bovine serum, 1% penicillin/streptomycin). Cells were incubated at 37 °C in a humidified atmosphere containing 5% CO_2_ for 24 h to allow cells to adhere to the plate. The media was replaced with fresh media 1 h prior to the addition of 100 μL of dithioether PC (final concentration ranged from 10 to 500 μg mL^–1^). The cytotoxicity of Lipofectamine 2000 (Invitrogen, Carlsbad, CA) was also tested at a final concentration from 10 to 100 μg mL^–1^. The cells were incubated with the formulations for 24 h and 5 μL of propidium iodide (PI) solution (eBioscience, San Diego) was added to each well. The cells were incubated with PI for 1 h at 37 °C in a humidified atmosphere containing 5% CO_2_ protected from light. Phase contrast and PI fluorescence images were acquired using fluorescence microscopy to determine cell viability.

## Results and discussion

**Scheme 1:**
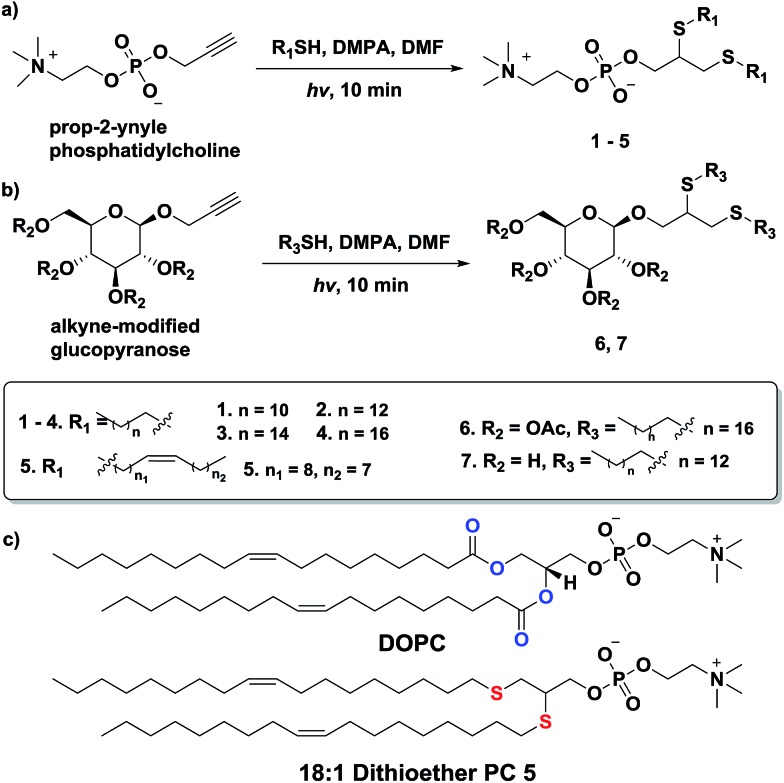
Rapid synthesis of dithioether phospholipids and glycolipids through photo-initiated thiol-yne click chemistry.

The mechanism of thiol-yne click chemistry has been previously elucidated.^52^ The addition of a thiyl radical to an alkyne results in a carbon-centered radical, which then subsequently abstracts a hydrogen from another thiol to form a vinyl sulfide moiety, regenerating another thiyl radical. The vinyl sulfide further reacts with the second thiyl radical to complete the reaction. The resulting alkyl thioethers are analogous to the two acyl chains of natural lipids (Scheme 1c). The thioether linkage is also a close mimic of the natural ether linkage found in many phospholipids.^53^ However, it should be mentioned that the dithioether lipids generated from thiol-yne chemistry are non-stereospecific. To prepare dithioether PCs, the alkynyl head group was combined with two equivalents of long-chain thiols and illuminated with UV light (354 nm) for 10 min, in the presence of radical initiator DMPA in DMF (Scheme 1a). Typical isolated yields of over 90% were achieved for dithioether PC preparation. By utilizing different alkyl thiols, dithioether PCs with different tail lengths were easily prepared. We synthesized dithioether PC **1–5**, to resemble natural diacylglycerol PC 1,2-dilauroyl-*sn*-glycero-3-phosphocholine (C12:0, DLPC), 1,2-dimyristoyl-*sn*-glycero-3-phosphocholine (C14:0, DMPC), DPPC (C16:0), 1,2-distearoyl-*sn*-glycero-3-phosphocholine (C18:0, DSPC), DOPC (C18:1, Δ9-*cis*), respectively.

In addition to accessing dithioether PCs with various tail lengths, the hydrophilic head group can be modified by using different alkyne precursors. Monosaccharide lipids are a class of lipids with polar sugar head groups, and are ubiquitous in nature.^54–56^ Galactocerebroside, an abundant glycolipid in the myelin bilayer, is synthesized by Schwann cells in the peripheral nervous system during myelination.^54^ Additionally, monogalactosyldiacylglycerol (MGDG) serves as a membrane lipid in the chloroplast of many plants and in bacteria that are in a phosphorus poor environment, helping to conserve phosphate for essential biochemical processes^55,56^ To determine if thiol-yne chemistry is suitable for synthesizing monosaccharide lipids, we reacted an alkyne modified glucopyranose to various alkyl thiols. We found that alkynyl-glucopyranose readily photoreacts with octanedecanethiol and tetradecanethiol under the same conditions as used for synthesizing dithioether PCs, to respectively to afford glycolipid analogs **6** (79% yield) and **7** (85% yield) (Scheme 1b). A major challenge when synthesizing glycolipids using conventional chemistries is the typical requirement of protection and deprotection for the complex sugar functional groups.^57^ Interestingly, the absence of protecting groups on the monosaccharide did not appear to interfere with the formation of lipid, likely due to the exquisite chemoselectivity of the thiol-yne chemistry. The high modularity and the flexibility of the thiol-yne click chemistry should allow facile access to many natural lipid analogs by combining appropriate alkynyl head groups and alkyl thiols.

**Fig. 1:**
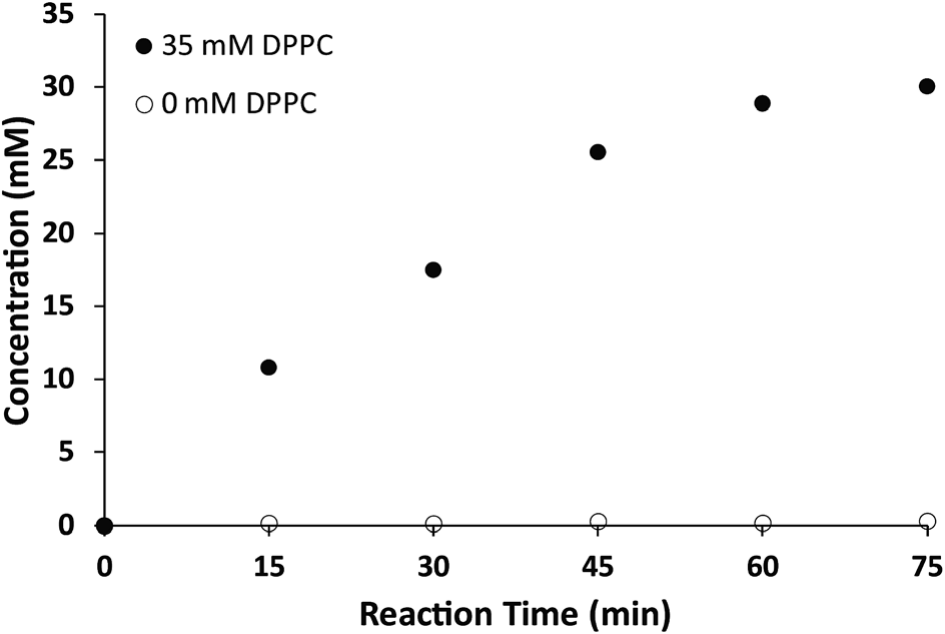
Formation of **1** in aqueous solution containing 35 mM DPPC. Formation of **1** slows as products saturate the solution. Only a trace amount of **1** was detected in the absence of DPPC. Data from a single collection set.

Additionally, we explored the *in situ* synthesis of dithioether phospholipids in aqueous solution. Replacing DMF with water severely decreased the yield of dithioether PCs. Recent elegant work has shown that micelle-forming amphiphiles can promote the formation of new single chain thioether amphiphiles by thiol-ene chemistry.^58^ We similarly found that addition of DPPC in the aqueous solvent allowed water insoluble dodecanethiol to interact with other water soluble precursors, driving the formation of dithioether PC product *in situ*. The progression of C12:0 dithioether PC **1** formation was monitored by LCMS-ELSD (Fig. S1†). The formation of dithioether PC product eventually slows after 75 min as it saturates the solution, at which point the formation of disulfide side product increases. As mentioned, only a trace amount of dithioether PC formed in the absence of DPPC (Fig. 1). The phospholipid promoted synthesis of dithioether PC suggests that autocatalytic formation of dithioether PC vesicles may be possible.^59^

**Fig. 2:**
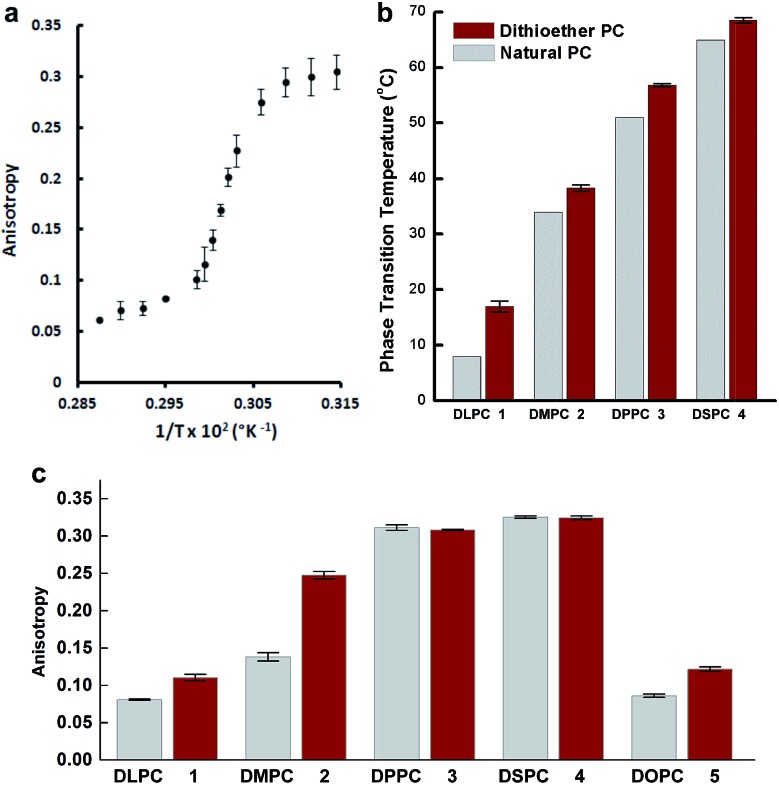
The *T*_C_ of dithioether PCs determined by steady-state anisotropic measurements. (a) Anisotropic curve of C18:0 dithioether PC **4**, a sudden change of slope indicates the membrane transitions from a gel state to a liquid state at 59 °C. (b) The *T*_C_ of C12:0, C14:0, C16:0 and C18:0 dithioether PC **1–4** are 7 °C, 28 °C, 47 °C and 59 °C, respectively, slightly higher than –2 °C, 24 °C, 41 °C and 55 °C of their natural PC analogs. (c) DPH anisotropy of dithioether PC **1–5** and their natural analogs at 23 °C. Error bars denote standard deviation (*n* = 3).

While the chemical structure of the dithioether PC resembles that of diacylglycerol PC, we next sought to characterize their functional similarity. Natural phospholipids and glycolipids readily form bilayer membranes when hydrated. We found that all lipids synthesized also formed membranes when thin films were hydrated. An important property of glycerolipid membranes is their fluidity and the gel phase to liquid disordered phase transition temperature (*T*_C_). These parameters largely depend on the nature of the head group and the length and composition of the alkyl chains. We were thus interested in determining how dithioether PCs compared with respect to their diacylglycerolipid equivalents. To determine the *T*_C_ of the dithioether PC, steady-state anisotropy was employed. Small unilamellar vesicle samples (SUV) with a diameter of 100 nm were prepared by sonication of dry lipid film, followed by extrusion and incubation with membrane fluorescent probe DPH. The anisotropy measurements were plotted as a function of temperature (Fig. 2a and S2†). The *T*_C_ is the temperature at which the greatest slope on the anisotropic curve is observed. The steady-state anisotropy measurements indicated that the *T*_C_ of the dithioether PC are approximately 4–10 °C higher than their diacylglycrol PC (Fig. 2b).^60^ Previous studies on C16:0 diether PC, 1,2-dihexadecyl-*glycero*-3-phosphocholine, show a slight increase in *T*_C_ as compared to DPPC (45 °C *vs.* 41 °C).^60,61^ The observed elevation of *T*_C_ in C16:0 diether PC is likely due to the absence of carboxyl groups in the region of the glycerol backbones, allowing for a more ordered packing in the plane of the lipid bilayer.^61^ The same rationale can be applied to the dithioether PC, where a change from oxygen to larger sulfur atoms presumably increases the lipophilicity, leading to a tighter packing of lipids within the membrane.^62^ Similarly, the steady-state anisotropic measurements of dithioether PCs were also measured at the room temperature, with higher values indicating a reduced membrane fluidity. We found that membranes consisting of **1**, **2** or **5** all exhibited reduced membrane fluidity compared to the corresponding diacylglycerol PC analogs. At 23 °C, the anisotropy measurements for **3** and **4** are similar to their natural analogs DPPC and DSPC, presumably because the *T*_C_ of long chain saturated phospholipids are much higher than 23 °C, and therefore all lipids will be in the ordered gel phase (Fig. 2c). The dithioether PCs with longer saturated carbon chains (**3** and **4**) exhibit a sharp drop in anisotropy during the phase transition, indicating a smaller melting range for the membrane. This phenomenon is likely related to the better phase transition cooperativity of the lipid membrane. In contrast, the increased melting ranges for shorter carbon chain lipids (**1** and **2**) suggest that these lipids are less cooperative (Fig. S2†). The observation of decreased phase transition cooperativity in lipids with shorter alkyl tails is similar to previous studies.^63^ We were not able to determine the *T*_C_ of **5** with the lowest temperature setting of our instrument (–10 °C), which is not surprising since a similar phospholipid (DOPC) has a *T*_C_ at –17 °C.^60^

**Fig. 3:**
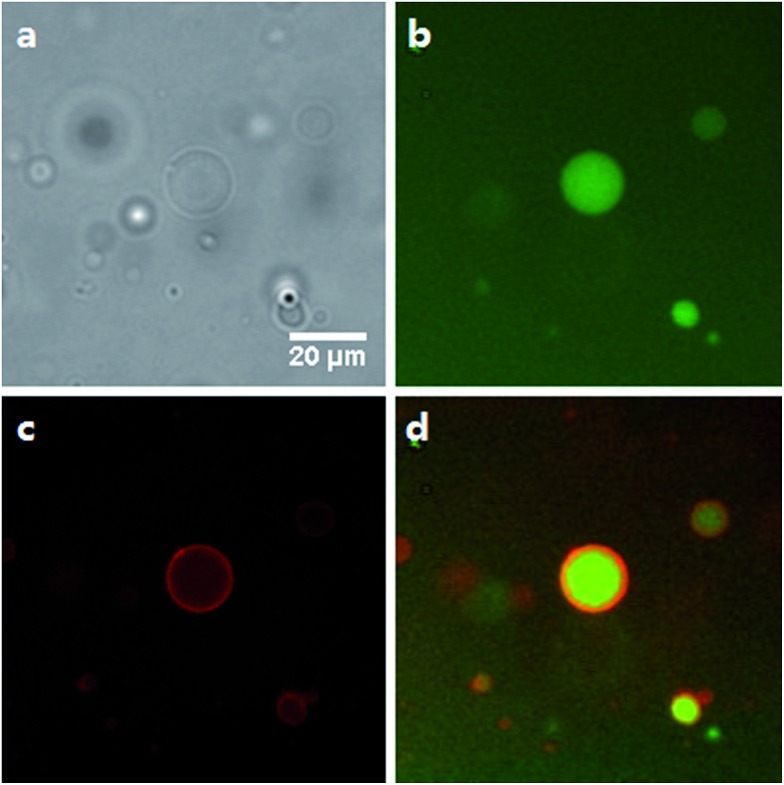
Fluorescence microscopy images of GUVs consisting of 100% C14:0 dithioether PC **2**. (a) Brightfield. (b) GFP channel illustrating HPTS encapsulation. (c) DesRed channel illustrating the staining of the lipid membrane with Texas Red DHPE. (d) Merge of GFP and DesRed channels.

Giant unilamellar vesicles (GUVs) are important model systems for imitating biological membranes due to their comparable size to living cells and the ability of GUVs to function as a physical barrier for encapsulated contents. GUVs made of dithioether PC were prepared using a water-in-oil emulsion technique^51^ and they were observed by fluorescence microscopy with encapsulated HPTS as an aqueous fluorescent marker. Texas Red DHPE was also used to visualize the lipid membrane (Fig. 3, S3 and S4†). Short chain lipids **1**, **2** and unsaturated lipid **5** readily formed GUVs at room temperature, probably because of their relatively low *T*_C_.^64^

The compatibility of dithioether PC with GUV formation methods allows the formation of biomimetic membranes. As a model system for primitive living cells, liposomes are excellent biocompatible microreactors for mimicking cellular processes.^65^ Previous studies have reported that functional proteins can be expressed in liposomes consisting of natural phospholipids using cell free expression systems.^44,66^ In order to examine the biocompatibility of the dithioether lipids with these systems, we prepared GUVs with encapsulated *E. coli* cell lysate and plasmid DNA encoding GFP by utilizing the same water-in-oil emulsion technique. After incubating at 37 °C for 4 hours, fluorescence microscopy revealed that functional GFP was successfully expressed in the GUVs (Fig. S5†). The expression of GFP demonstrates that the dithioether PC are capable of sustaining essential cellular biochemistry such as transcription and translation. Dithioether PCs could serve as alternative lipids for forming stable liposomes or micro-bioreactors.

**Fig. 4:**
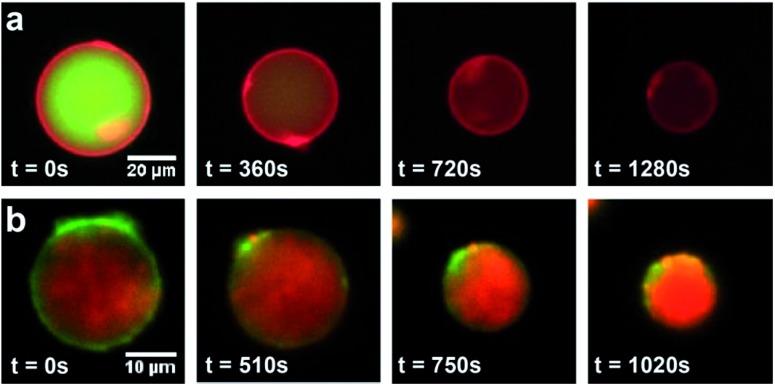
Time-lapse fluorescence microscopy images of shrinking vesicles. (a) The GFP leaked from DOPC/**5** GUV upon the shrinkage of the vesicle by PLA_2_ treatment. The GUV membrane was stained with Texas Red DHPE. (b) The 100 nm red fluorescent sulfate-modified polystyrene nanoparticles were contained and concentrated in DOPC/**5** GUV as the vesicle shrank. The membrane was stained with NBD-PE.

Dithioether PCs are unnatural lipid analogs capable of forming membranes. A potential advantage of such lipids is enhanced stability to enzymes that normally cleave diacylglycerol phospholipids. The PLA_2_ superfamily consists of a group of enzymes that hydrolyze diacylglycerol phospholipids at the *sn*-2 acyl bond, forming lysolipids and fatty acids.^67,68^ They are commonly found in mammalian tissue and function in the venom of bee and viper snakes by promoting cell lysis and causing inflammation.^67^ Dithioether PCs lack ester bonds and are therefore stable in the presence of PLA_2_. Enzymatic assays showed that bee venom PLA_2_, which fully hydrolyzes DOPC to yield C18:1 lysolipid and oleic acid, has no hydrolytic activity against a C18:1 dithioether PC substrate **5** under the same conditions (Fig. S6 and S7†). Further, PLA_2_ had no effect on the membrane size and integrity of the dithioether GUVs (Fig. S6†). Previous work has demonstrated that removal of diacylglycerol phospholipids from vesicle bilayers leads to liposome shrinkage.^69,70^ One potential application for vesicle size reduction may be to increase encapsulated drug^45^ and nanoparticle^71^ concentrations, a feature that could aid drug and imaging agent delivery. GUV shrinkage studies were previously reported by removal of diacylglycerol phospholipids *via* hydrolysis using phospholipases.^69,70^ However, this method leads to a complete hydrolysis of the vesicle, making it not feasible for liposome preparation or concentration of encapsulated material. The dithioether PCs are capable of resisting phospholipase hydrolysis. Therefore, we formulated GUVs with a mixture of natural and dithioether PC. Upon exposure of vesicles to PLA_2_, the diacylglycerol PCs are hydrolyzed, and removed from the membrane while the phospholipase immune dithioether PC are retained. This simple approach of selectively removing diacylglycerol phospholipid by PLA_2_ hydrolysis significantly reduced the size and volume of the GUVs. GUVs (85 mol% DOPC, 15 mol % **5**) were treated with 6.7 μg mL^–1^ bee venom PLA_2_ in 100 mM HEPES buffer containing 10 mM CaCl_2_ and 35 mM KCl at pH = 7.4, and they gradually shrank from a diameter of 47.5 μm to 25 μm. During the shrinkage, encapsulated GFP leaked out of the liposome (Fig. 4a and video S1 in the ESI†). Although the protein GFP readily leaked out of the vesicles during the vesicle shrinkage, larger nanoparticles were contained and concentrated. GUVs with the same lipid contents encapsulating 100 nm red fluorescent sulfate-modified polystyrene nanoparticles were prepared. Upon treatment with PLA_2_, the GUV also gradually shrank to nearly 50% of its original diameter and 30% of its original volume. The interior fluorescence became brighter, demonstrating that the nanoparticles were concentrated (Fig. 4b and video S2 in the ESI†). Importantly, the shrunk vesicles were stable after the treatment of phospholipase. This approach allows for tuning the degree of GUV shrinkage by introducing a different fraction of phospholipase resistant lipids. It may be further utilized for concentrating larger biomolecular complexes such as ribosomes, genomic DNA, or nanomaterials with therapeutic or imaging applications.^72–74^

**Fig. 5:**
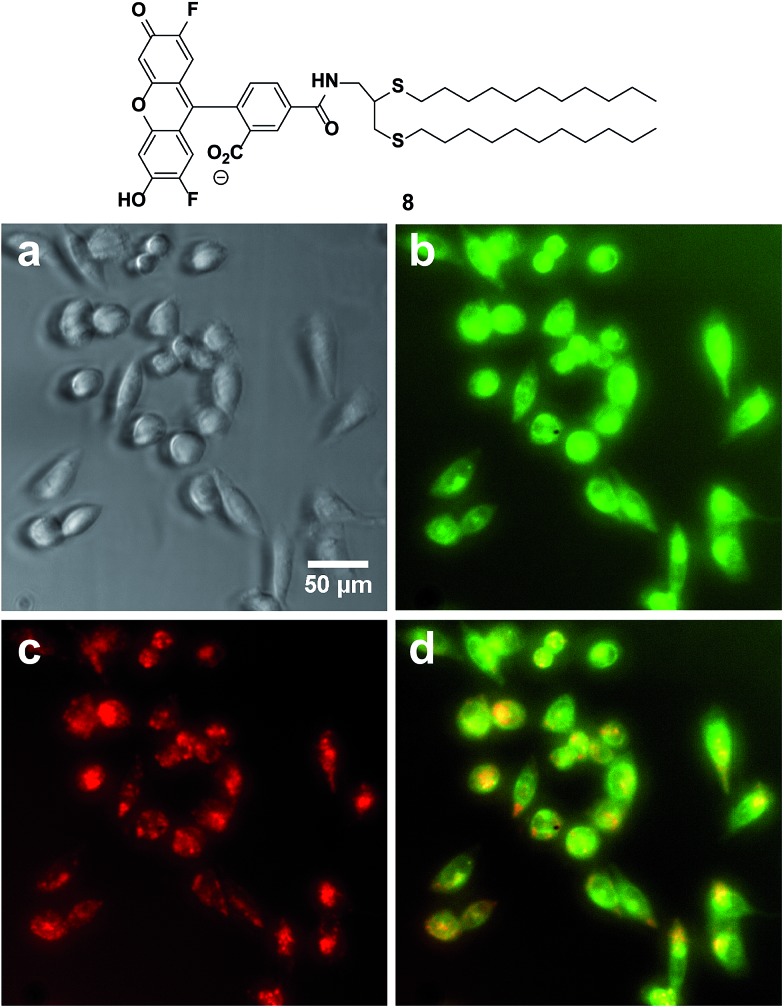
Localization of fluorescent dithioether lipid **8** in live Chinese hamster ovary cells. (a) Phase contrast (b) GFP channel illustrating the dispersion of **8** in cell membranes. (c) DesRed channel illustrating the dispersion of Texas Red DHPE in cell membranes. (d) Merge of GFP and Texas Red Channel.

Fusogenic liposomes containing fluorescent lipid moieties, such as Texas Red DHPE, are known to widely disperse in the cell membrane.^75^ In order to determine the localization of dithioether lipids in living cells, a fluorescently labelled C12:0 dithioether lipid **8** containing an Oregon Green fluorophore moiety was synthesized. The cells were incubated with 5 μM of **8** and 5 μM Texas Red DHPE for 1 h under 5% CO_2_ and 95% humidity. Similar to the Texas Red DHPE, **8** was widely dispersed in the cell membrane (Fig. 5).

**Fig. 6:**
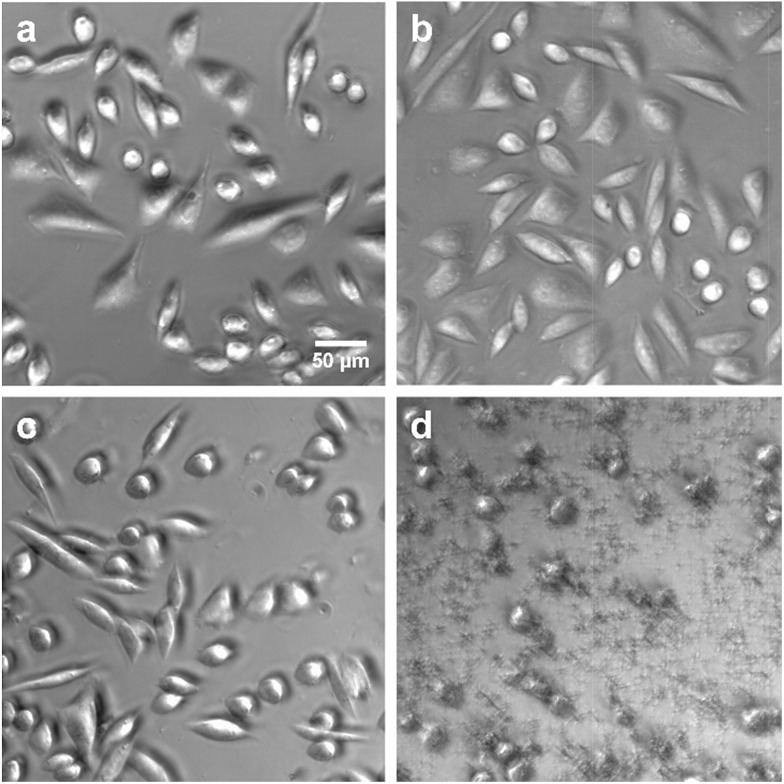
Phase contrast images of CHO cells after (a) no addition of lipid (b) addition of 500 μg mL^–1^ POPC (c) addition of 500 μg mL^–1^ **5** (d) addition of 50 μg mL^–1^ Lipofectamine 2000. After 24 h of incubation, cells in a, b and c were alive and adherent to the surface. Cells in d were dead as they were no longer adherent to the surface.

To apply dithioether lipids for biomedical applications such as liposome mediated drug delivery, it is important that the lipids show minimal toxicity to living cells. To better understand the potential cytotoxicity of the dithioether PCs to living cells, lipid **5** was exposed to Chinese hamster ovary (CHO) cells. **5**, at the range of the 10–500 μg mL^–1^ concentration, was incubated with CHO cells under 5% CO_2_ and 95% humidity for 24 h. Cells treated with 500 μg mL^–1^ **5** were fully viable when their membrane integrity was assayed by the DNA staining dye propidium iodide (PI).^76,77^ When PI was incubated with the cells for 1 h, no increase in fluorescence was observed, suggesting the cell membrane was intact (Fig. S8†). The same results were obtained for control experiments (no lipid addition) and the addition of the nontoxic natural lipid POPC. Conversely, cells treated with 50 μg mL^–1^ Lipofectamine 2000 mostly died as the cells were no longer adherent and bright PI fluorescence was observed, suggesting the loss of membrane integrity (Fig. 6).

The limited toxicity of the dithioether lipid opens up a variety of promising future biochemical and biomedical applications. For instance, it is worth mentioning the possible application of dithioether PC as synthetic lung surfactants. Exogenous lung surfactant, which primarily contains PCs and traces of proteins and other substances, are often replaced in premature infants to avoid alveolar collapse by maintaining a low surface tension.^9^ Current lung surfactants made of diacylglycerol PCs can be degraded by phospholipases during inflammatory injury.^78–80^ The introduction of synthetic dithioether PCs would not only prevent the degradation of the active ingredients in the lung surfactants, but also prevent the generation of a large amount of lysolipid and fatty acids that could interfere with normal cellular activities. Phospholipase resistant phospholipids analogs, such as diether phospholipids and phosphonolipids, have been tested to have very high surface activity.^46,81–83^ Therefore, we envision that the facile synthesis and the phospholipase resistance property of the dithioether phospholipids would greatly benefit the research and development of a novel and safer lung surfactant.

## Conclusions

In summary, we have developed a novel class of dithioether phospho- and glycolipids with various alkyl tail lengths through thiol-yne click chemistry. Thiol-yne click chemistry provides a powerful tool that greatly expands the phospho- and glycolipid arsenal for the application of developing liposomal drug delivery systems, novel phospholipid material for lung surfactant, bioreactors, and artificial cells. In addition, the orthogonality of the reaction and facile one-step synthesis allow the easy scale-up of the reaction, ideal for practical materials applications. We have determined the phase transition temperatures of the saturated dithioether PCs, which could be a useful reference for the application of dithioether phospholipids and its analogs. We have demonstrated the biocompatibility and safety of the synthetic lipid, as dithioether lipids can host cell-free expression systems and are nontoxic to human cells. The dithioether PC resistance against phospholipase A_2_ allows the controlled shrinkage of mixed dithioether phospholipid/DOPC GUVs and the concentration of encapsulated nanomaterials. It may further allow the concentration of large protein and protein–DNA complexes, nanoparticles and polymersomes.
