# Supplementary material for: Rapid access to phospholipid analogs using thiol-yne chemistry
Source: Chem Sci. 2015 May 19;6(7):4365–72. doi: 10.1039/c5sc00653h (PMC5707512; doi:10.1039/c5sc00653h)
Supplement: Supplementary file 1 [file SC-006-C5SC00653H-s001.pdf]

Electronic Supplementary Material (ESI) for Chemical Science.
This journal is © The Royal Society of Chemistry 2015

Rapid Access to Phospholipid Analogues Using Thiol-Yne Chemistry

Cun Yu Zhou‡, Haoxing Wu‡, Neal K. Devaraj*

Department of Chemistry and Biochemistry, University of California, San Diego, La Jolla, California 92093

Supplementary Information

1. Characterization Data for Dithioether Phosphatidylcholine **2-5** S2
   1.1 Synthesis of C14:0 Dithioether PC **2** S2
   1.2 Synthesis of C16:0 Dithioether PC **3** S2
   1.3 Synthesis of C18:0 Dithioether PC **4** S2
   1.4 Synthesis of C18:1 Dithioether PC **5** S2
2. General Procedure for Synthesis of Dithioether Glucopyranosyl Lipid S3
   2.1 Synthesis of Acetyl-Protected Glucopyranosyl Lipid **6** S3
   2.2 Synthesis of Protection-Free Glucopyranosyl Lipid **7** S3
3. Synthesis of Oregon Green C12:0 Dithioether Lipid S4
4. HPLC Traces for Lipid Driven Formation of Dithioether PC S5
5. Dithioether PC Anisotropy Measurements S6
6. Fluorescence Microscopy Images of Giant Unilamellar Vesicles Formation S7
7. Cell-Free Expression of GFP in Dithioether PC GUVs S8
8. Dithioether PC GUV Stability against PLA_2 S9
9. HPLC Analysis of Dithioether PC Stability against PLA_2 S10
10. Live Cell Imaging S11
11. Cell Viability Study S11
12. Supporting Information Videos S12
13. NMR Spectra for Synthesized Material S13
   13.1 C12:0 Dithioether PC **1** S13
   13.2 C14:0 Dithioether PC **2** S15
   13.3 C16:0 Dithioether PC **3** S17
   13.4 C18:0 Dithioether PC **4** S19
   13.5 C18:1 Dithioether PC **5** S21
   13.6 Acetyl-Protected Glucopyranosyl Lipid **6** S23
   13.7 Protection-Free Glucopyranosyl Lipid **7** S25
   13.8 Oregon Green C12:0 Dithioether Lipid **8** S27
14. Supporting Information References S28

1. Characterization Data for Dithioether Phosphotidylcholine 2-5

1.2 Synthesis of C14:0 dithioether PC **2**: 11 mg of the starting material prop-2-ynyle phosphatidylcholine[1] yields 32 mg **2** as a white solid, in 95% yield. ^{1}H NMR (500 MHz, $CDCl_3$) δ 4.32 (s, 2H), 4.09 (s, 1H), 3.92 (s, 1H), 3.81 (s, 2H), 3.37 (s, 9H), 2.98 – 2.91 (m, 1H), 2.90 – 2.83 (m, 1H), 2.80 – 2.73 (m, 1H), 2.55 (dd, *J* = 15.0, 7.2 Hz, 4H), 1.63 – 1.49 (m, 4H), 1.34 (s, 4H), 1.24 (s, 40H), 0.87 (t, *J* = 6.8 Hz, 6H). ^{13}C NMR (126 MHz, $CDCl_3$) δ 76.70, 66.48, 59.45, 54.53, 54.53, 54.53, 46.47, 34.52, 33.24, 32.07, 32.07, 31.70, 30.14, 29.97, 29.88, 29.88, 29.88, 29.88, 29.88, 29.88, 29.85, 29.85, 29.83, 29.83, 29.80, 29.80, 29.52, 29.52, 29.52, 29.52, 29.23, 29.18, 22.84, 22.84, 14.28, 14.28. HRMS $[M+Na]^+$ m/z calcd. for $[C_{36}H_{76}NO_4PS_2Na]^+$ 704.4846, found 704.4848.

1.3 Synthesis of C16:0 dithioether PC **3**: 11 mg of the starting material prop-2-ynyle phosphatidylcholine yields 37 mg **3** as a white solid, in 97 % yield. ^{1}H NMR (500 MHz, $CDCl_3$) δ 4.31 (s, 2H), 4.07 (s, 1H), 3.91 (s, 1H), 3.81 (s, 2H), 3.38 (s, 9H), 2.97 – 2.89 (m, 1H), 2.88 – 2.83 (m, 1H), 2.79 – 2.73 (m, 1H), 2.53 (dd, *J* = 15.7, 8.0 Hz, 4H), 1.60 – 1.46 (m, 4H), 1.34 (s, 4H), 1.24 (s, 48H), 0.86 (t, *J* = 7.0 Hz, 6H). ^{13}C NMR (126 MHz, $CDCl_3$) δ 76.70, 66.47, 59.45, 54.53, 54.53, 54.53, 46.44, 34.47, 33.21, 32.07, 32.07, 31.70, 30.15, 29.98, 29.91, 29.91, 29.91, 29.91, 29.91, 29.91, 29.89, 29.89, 29.89, 29.89, 29.89, 29.89, 29.83, 29.83, 29.83, 29.83, 29.54, 29.54, 29.53, 29.53, 29.25, 29.21, 22.84, 22.84, 14.28, 14.28. HRMS $[M+Na]^+$ m/z calcd. for $[C_{40}H_{84}NO_4PS_2Na]^+$ 760.5472, found 760.5473.

1.4 Synthesis of C18:0 dithioether PC **4**: 25 mg of the starting material prop-2-ynyle phosphatidylcholine yields 83 mg **4** as a white solid, in 91% yield. ^{1}H NMR (500 MHz, $CDCl_3$) δ 4.30 (s, 2H), 4.08 (s, 1H), 3.96 (s, 1H), 3.79 (s, 2H), 3.37 (s, 9H), 2.98 – 2.89 (m, 1H), 2.88 – 2.83 (m, 1H), 2.79 – 2.73 (m, 1H), 2.61 – 2.45 (m, 4H), 1.60 – 1.46 (m, 4H), 1.34 (s, 4H), 1.24 (s, 56H), 0.86 (t, *J* = 7.0 Hz, 6H). ^{13}C NMR (126 MHz, $CDCl_3$) δ 76.70, 66.50, 59.44, 54.52, 54.52, 54.52, 46.50, 34.56, 33.28, 32.06, 32.06, 31.69, 30.13, 29.97, 29.89, 29.89, 29.89, 29.89, 29.89, 29.89, 29.89, 29.89, 29.86, 29.86, 29.86, 29.86, 29.86, 29.86, 29.81, 29.81, 29.81, 29.81, 29.80, 29.80, 29.51, 29.51, 29.51, 29.51, 29.22, 29.17, 22.83, 22.83, 14.27, 14.27. HRMS $[M+Na]^+$ m/z calcd. for $[C_{44}H_{92}NO_4PS_2Na]^+$ 816.6098, found 816.6099.

1.5 Synthesis of C18:1 dithioether PC **5**: 8 mg of the starting material prop-2-ynyle phosphatidylcholine yields 17.6 mg **5** as a waxy solid, in 61 % yield. ^{1}H NMR (500 MHz, $CDCl_3$) δ 5.37 (s, 3H), 5.34 (s, 1H), 4.34 (s, 2H), 4.08 (s, 1H), 3.94 (s, 1H), 3.81 (s, 2H), 3.39 (s, 9H), 3.01 – 2.93 (m, 1H), 2.89 (dd, *J* = 13.1, 6.6 Hz, 1H), 2.78 (dd, *J* = 13.2, 6.0 Hz, 1H), 2.54 (dd, *J* = 15.8, 8.2 Hz, 4H), 1.97 (dt, *J* = 11.7, 6.6 Hz, 8H), 1.58 – 1.51 (m, 4H), 1.34 – 1.21 (m, 44H), 0.87 (t, *J* = 7.0 Hz, 6H). ^{13}C NMR (126 MHz, $CDCl_3$) δ 130.55, 130.37, 130.09, 129.90, 76.72, 66.66, 59.40, 54.71, 54.71, 54.71, 46.58, 34.67, 33.37, 32.78, 32.78, 32.05, 32.05, 31.71, 31.13, 30.12, 29.97, 29.94, 29.92, 29.83, 29.83, 29.81, 29.81, 29.68, 29.65, 29.65, 29.63, 29.48, 29.48, 29.48, 29.48, 29.46, 29.46, 29.36, 29.36, 29.34, 29.34, 29.22, 29.16, 27.37, 22.83, 22.83, 14.27, 14.27. HRMS $[M+Na]^+$ m/z calcd. for $[C_{44}H_{88}NO_4PS_2Na]^+$ 812.5785, found 812.5784.

2. General Procedure for Synthesis of Dithioether Glucopyranosyl Lipid

In a 10 mL microwave reaction tube, the alkyne-modified glucose and alkylthiol were sonicated under N_2 protection for 60 min to dissolve in 3 mL DMF. The mixture was then transferred to a 3500 μL macro fluorescence cuvette and 2,2-dimethoxy-2-phenylacetophenone (2 mg, 0.008 mmol) was added. The reaction proceeded under UV (354 nm) for 10 minutes. The solvent was removed and the residue was purified by column chromatography.

2.1 Synthesis of Acetyl-Protected Glucopyranosyl Lipid **6**: 40 mg of the starting material 2-propynyl-tetra-*O*-acetyl-β-D-glucopyranoside (Sigma-Aldrich, St. Louis, MO) affords 78 mg **6** as white solid after silica column chromatography (DCM : MeOH = 20 : 1). Yield: 79%. ^{1}H NMR (500 MHz, $CDCl_3$) δ 5.19 (td, *J* = 9.5, 3.2 Hz, 1H), 5.07 (td, *J* = 9.7, 4.0 Hz, 1H), 4.99 (ddd, *J* = 9.7, 8.0, 6.4 Hz, 1H), 4.52 (d, *J* = 8.0 Hz, 1H), 4.26 (dd, *J* = 12.3, 4.7 Hz, 1H), 4.15 – 4.03 (m, 2H), 3.73 – 3.57 (m, 2H), 2.91 (ddd, *J* = 13.3, 7.2, 5.2 Hz, 1H), 2.77 (dtd, *J* = 17.8, 13.3, 6.7 Hz, 2H), 2.56 – 2.48 (m, 4H), 2.08 (s, 3H), 2.05 (d, *J* = 2.6 Hz, 3H), 2.01 (s, 3H), 1.99 (s, 3H), 1.57 – 1.52 (m, 4H), 1.37 – 1.32 (m, 4H), 1.22 (d, *J* = 18.3 Hz, 56H), 0.86 (t, *J* = 6.9 Hz, 6H). ^{13}C NMR (126 MHz, $CDCl_3$) δ 179.80, 170.79, 170.42, 170.39, 169.54, 169.52, 169.43, 169.38, 101.38, 101.05, 72.87, 72.76, 71.92, 71.87, 71.59, 71.24, 71.23, 68.43, 61.99, 45.76, 45.24, 34.60, 34.58, 33.54, 33.21, 32.05, 32.05, 32.02, 31.74, 30.03, 30.00, 29.87, 29.83, 29.83, 29.83, 29.83, 29.83, 29.83, 29.83, 29.83, 29.81, 29.81, 29.79, 29.79, 29.76, 29.75, 29.69, 29.69, 29.67, 29.49, 29.49, 29.42, 29.40, 29.39, 29.07, 29.03, 29.03, 22.82, 22.82, 20.91, 20.89, 20.75, 20.73, 14.27, 14.27. HRMS $[M+Na]^+$ m/z calcd. for $[C_{53}H_{98}O_{10}S_2Na]^+$ 981.6494, found 981.6488.

2.2 Synthesis of Protection-Free Glucopyranosyl Lipid **7**: 11 mg of the starting material 2-propynyl-β-D-glucopyranoside[2] affords 28.8 mg **7** as white solid after silica column chromatography (DCM : MeOH : H_2O = 20 : 1 : 0.1). Yield: 85%. ^{1}H NMR (500 MHz, $CDCl_3$) δ 4.87 (s, 1H), 4.67 (s, 1H), 4.33 (d, J = 7.5 Hz, 1H), 4.07 (ddd, *J* = 16.4, 10.3, 5.3 Hz, 1H), 3.84 (s, 2H), 3.74 (ddd, *J* = 17.0, 10.3, 5.9 Hz, 1H), 3.64 – 3.51 (m, 2H), 3.44 – 3.38 (m, 1H), 3.31 (dd, *J* = 9.0, 2.8 Hz, 1H), 3.01 – 2.96 (m, 1H), 2.92 – 2.76 (m, 2H), 2.56 (dt, *J* = 14.9, 7.5 Hz, 4H), 2.45 (s, 1H), 1.57 (dt, J = 15.0, 7.4 Hz, 4H), 1.35 (s, 4H), 1.31 – 1.19 (m, 40H), 0.87 (t, *J* = 6.9 Hz, 6H).^{13}C NMR (126 MHz, $CDCl_3$) δ 103.43;103.21, 76.34;76.29, 75.76, 73.53;73.47, 71.41;71.13, 69.60;69.52, 61.71;61.62, 45.76;45.63, 36.70, 34.82;34.69, 33.40;33.38, 32.08, 32.08, 31.64;31.62, 30.03;30.01, 29.89, 29.89, 29.89, 29.89, 29.89, 29.89, 29.86, 29.86, 29.84, 29.84, 29.79, 29.79, 29.54, 29.54, 29.52, 29.52, 29.23;29.21, 29.16, 22.85, 22.85, 14.29, 14.29. HRMS $[M-H]^-$ m/z calcd. for $[C_{37}H_{74}O_6S_2]^-$ 677.4854, found 677.4858.

3. Synthesis of Oregon Green C12:0 Dithioether Lipid

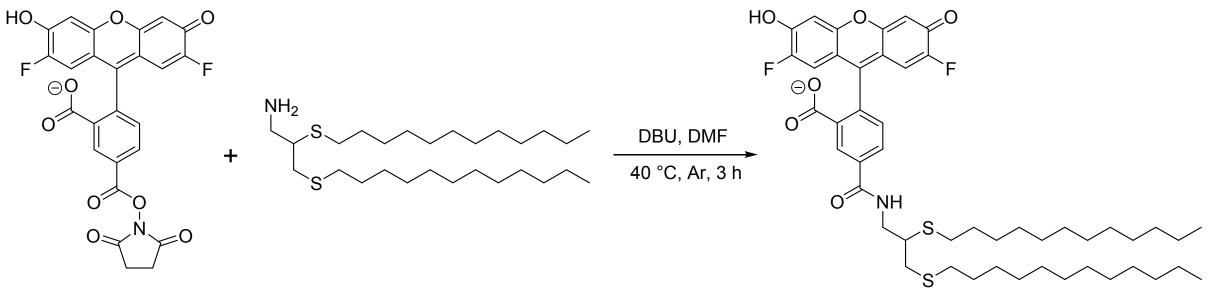

Synthesis of 5-((2,3-bis(dodecylthio)propyl)carbamoyl)-2-(2,7-difluoro-3-hydroxy-6-oxo-6,9a-dihydro-1*H*-xanthen-9-yl)benzoic acid **8**: 2,3-bis(dodecylthio)propan-1-amine[3] (2.3 mg, 5 µmol) was dissolved in 50 µL anhydrous DMF. DBU (1.2 mg, 7.5 µmol) was added to the mixture and allowed to stir at room temperature for 10 min. A solution of 50 µL of 50 mM Oregon Green 488 succiniymidyl ester (Molecular Probes, Eugene, OR) (1.3 mg, 2.5 µmol) in DMF was added. The mixture was stirred at 40 °C for 3 h under argon. DMF was removed *in vacuo* and the residue was purified by HPLC. 1.8 mg of 8 was isolated as an orange solid in 84% yield. ^{1}H NMR (500 MHz, Methanol-d_4) δ 8.48 (s, 1H), 8.22 (dd, *J* = 8.0, 1.4 Hz, 1H), 7.35 (d, *J* = 8.0 Hz, 1H), 6.84 (d, *J* = 7.4 Hz, 2H), 6.46 (d, *J* = 10.9 Hz, 2H), 3.78 (dd, *J* = 13.7, 6.5 Hz, 1H), 3.56 (dd, *J* = 13.7, 7.4 Hz, 1H), 3.16 (p, *J* = 6.9 Hz, 1H), 2.90 – 2.77 (m, 2H), 2.65 (td, *J* = 7.2, 2.8 Hz, 2H), 2.60 (t, *J* = 7.3 Hz, 2H), 1.65 – 1.55 (m, 4H), 1.46 – 1.34 (m, 4H), 1.25 (d, *J* = 6.5 Hz, 32H), 0.87 (td, *J* = 7.0, 2.6 Hz, 6H). HRMS $[M\text{-}H]^-$ m/z calcd. for $[C_{48}H_{64}F_2NO_6S_2]^-$ 852.4149, found 852.4144. HPLC gradient: 0 min – 2.5 min 85% *Phase B* in *Phase A*, 2.5 min – 18 min 85% *Phase B* in *Phase A* to 100% *Phase B*, 18 min – 28 min 100% *Phase B,* 28 min – 32 min 85% *Phase B* in *Phase A* (*Phase A*: H_2O with 0.1% formic acid, *Phase B*: MeOH with 0.1% formic acid).

4. HPLC Traces for Lipid Driven Formation of Dithioether PC

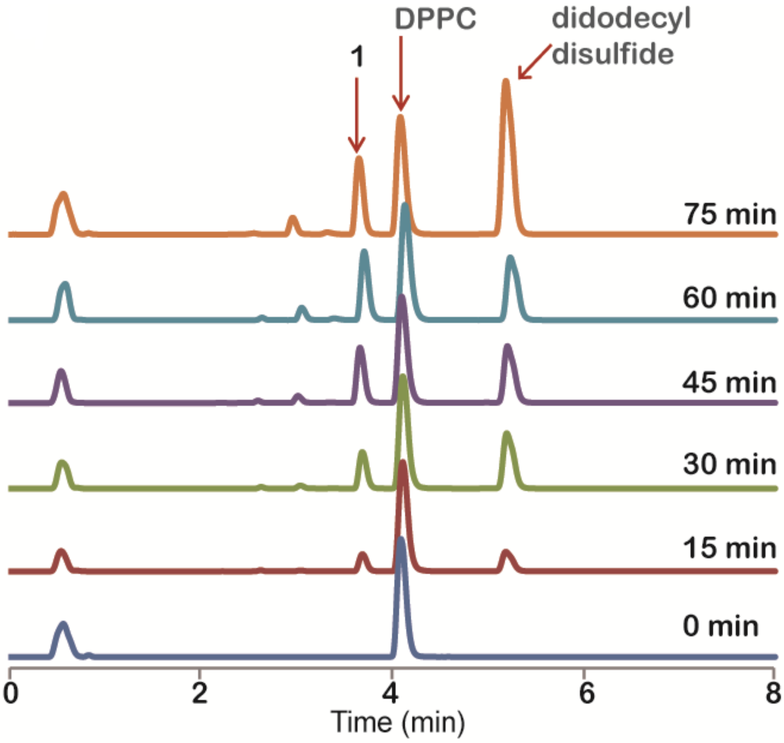

Figure S1. Progression of lipid **1** formation in 35 mM DPPC solution monitored by HPLC-ELSD. Concentration of **1** calculated by known standard calibration. The amount of DPPC addition is crucial for high conversion of dithioether phospholipid. When the DPPC concentration is lower than 25 mM, the conversion is slow. Prolonged exposure to the UV light generates disulfide byproduct thus halting the reaction. When the DPPC concentration is higher than 50 mM, the solution is crowded with DPPC thus limiting conversion. We have also observed addition of TCEP, a reducing agent, prevents this reaction.

4. Dithioether PC Anisotropy Measurements

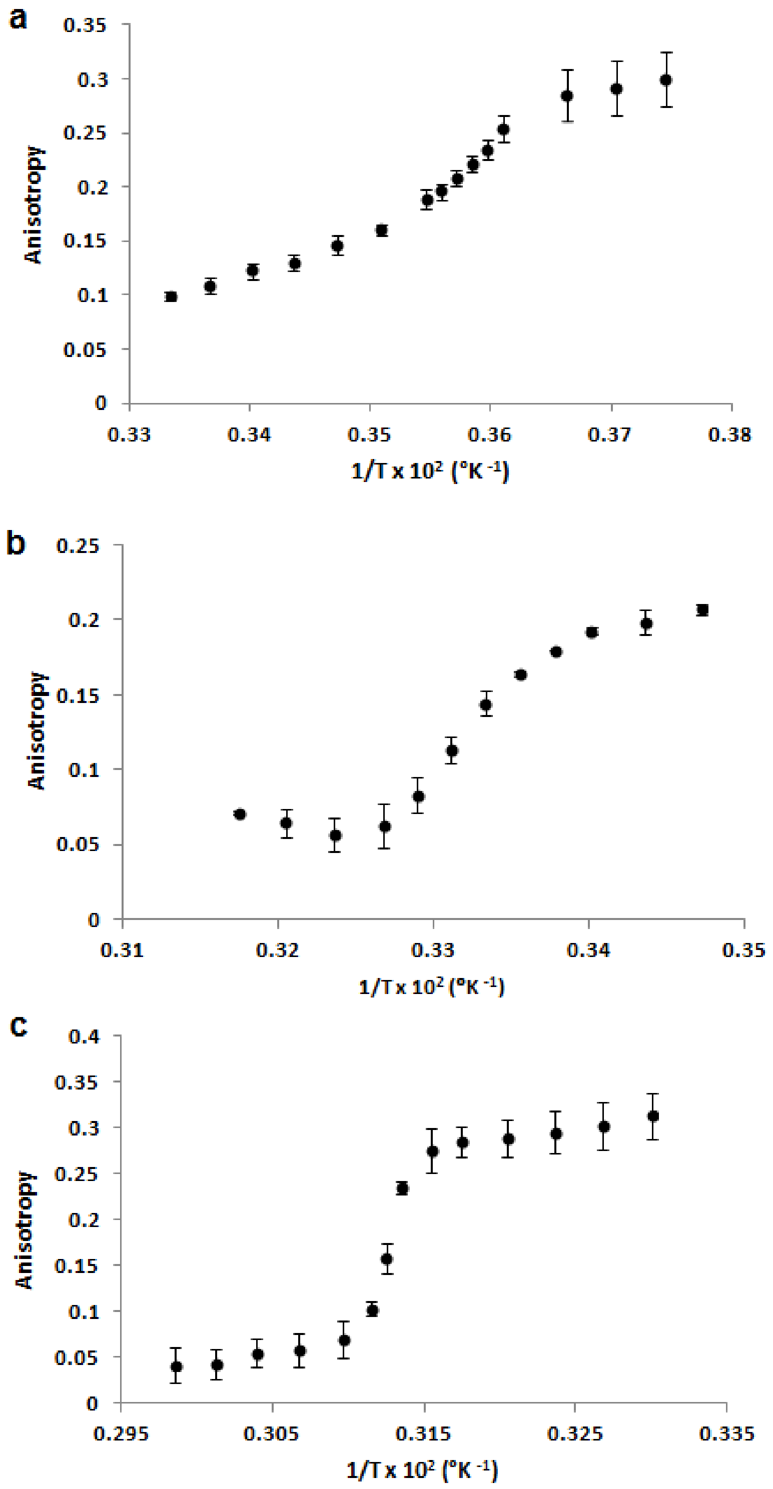

Figure S2. Steady-state fluorescence anisotropy curve as a function of temperature for saturated dithioether PCs. a) C12:0 lipid **1** b) C14:0 lipid **2** and c) C16:0 lipid **3**. Error bars denote SD of three measurements.

5. Fluorescence Microscopy Images of Giant Unilamellar Vesicles Formation

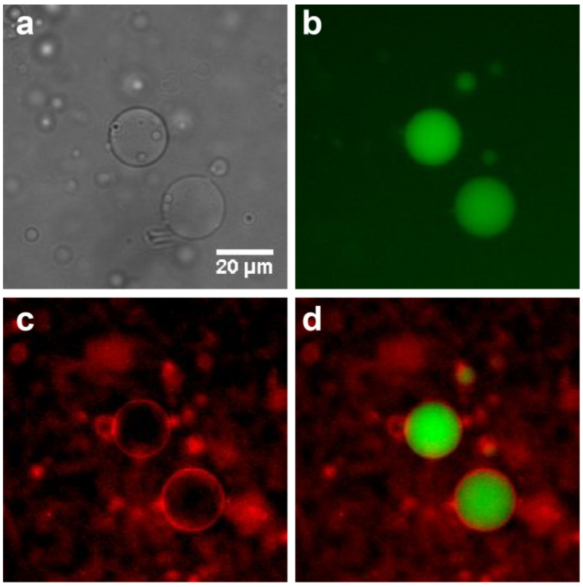

Figure S3. Fluorescence microscopy images of the GUVs made of C12:0 dithioether PC **1**. a) Brightfield. b) GFP channel illustrating HPTS encapsulation in the GUVs. c) DesRed channel illustrating the staining of the lipid membrane with Texas Red DHPE. d) Merge of GFP and DesRed channels.

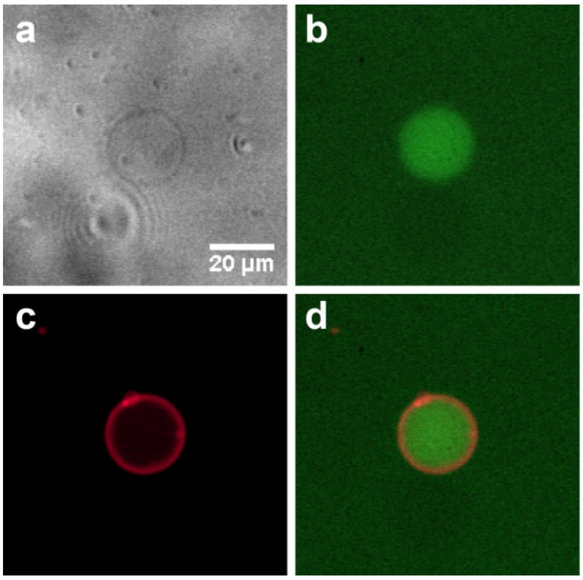

Figure S4. Fluorescence microscopy images of the GUVs made of C18:1 dithioether PC **5**. a) Brightfield. b) GFP channel illustrating HPTS encapsulation in the GUVs c) DesRed channel illustrating the staining of the lipid membrane with Texas Red DHPE. d) Merge of GFP and DesRed channels.

6. Cell-Free Expression of GFP in Dithioether PC GUVs

C18:0 dithioether PC **5** GUVs were formed with aforementioned procedure[4] In a 2 mL vial 40 µL of 10 mM **5** solution in chloroform was dried under N_2 to form a lipid film. 200 µL of light mineral oil was added to the vial and the mixture was sonicated for 1 hour until the lipid was fully dissolved in the oil. In a 0.7 mL eppendorf tube, 1 µg/50 µL pBEST-OR2-OR1-Pr-UTR1-deGFP-T500 Plasmid DNA[5] (Addgene, Cambridge, MA) in 10 µL *E. coli* cell lysates, S30 T7 high yield protein expression system (Promega, Madison, WI) was added to 100 µL of mineral oil containing lipid. The mixture was then flicked to form an emulsion. The emulsion was gently layered on top of 100 µL of lower buffer (100 mM HEPES, 200 mM glucose, in H_2O, pH = 7.4) in a different tube and the mixture was centrifuged at 10,000 rcf for 5 min. Light mineral oil was removed by vacuum suction and the vesicle solution was obtained. Upon the formation of GUVs, the sample was incubated at 37 °C for 4 h. 0.1 µL of 100 µM Texas Red DHPE solution in EtOH was added to 10 µL vesicle solution prior to microscope observation.

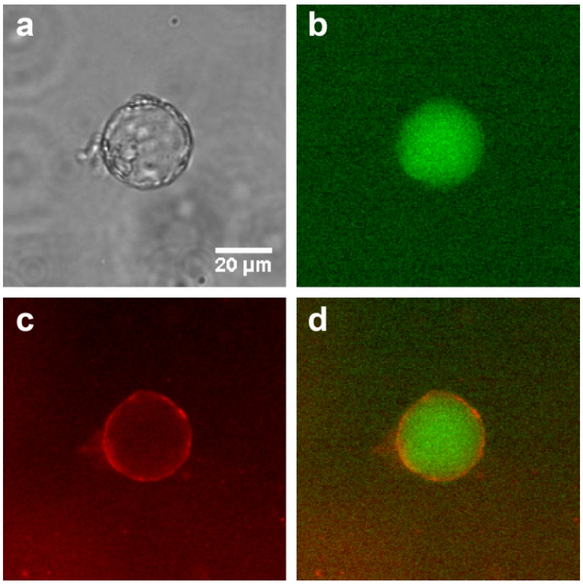

Figure S5. Fluorescence microscopy images of the GUVs made of C18:1 dithioether PC **5**. (a) Brightfield (b) GFP channel illustrating GFP expression after 4 h of incubation. (c) DesRed channel illustrating the staining of the lipid membrane with Texas Red DHPE. (d) Merge of GFP and DesRed channels.

7. Dithioether PC GUV Stability against PLA_2

GUVs were formed and characterized using aforementioned procedure[4]. In a 2 mL vial 40 μL of 10 mM lipid solution in chloroform was dried under N_2 to form a lipid film. 200 μL of light mineral oil was added to the vial and the mixture was sonicated for 1 hour until the lipid was fully dissolved in the oil. In a 0.7 mL Eppendorf tube 10 μL of the upper buffer (100 mM HEPES, 200 mM sucrose, 10 mM $CaCl_2$, 35 mM KCl, 1 mM HPTS in H_2O, pH = 7.4) was added to 100 μL of mineral oil containing lipid. The mixture was then flicked to form an emulsion. The emulsion was gently layered on top of 100 μL of lower buffer (100 mM HEPES, 200 mM glucose, 10 mM $CaCl_2$, 35 mM KCl in H_2O, pH = 7.4) in a different tube and the mixture was centrifuged at 10,000 rcf for 5 min. Light mineral oil was removed by vacuum suction and the vesicle solution was obtained. 0.1μL of 100 μM Texas Red DHPE solution in EtOH was added to 10 μL vesicle solution prior to microscope observation. Bee venom PLA_2 (Sigma-Aldrich, St. Louis, MO) was added to the samples to give a final concentration of 10 μg/mL. Fluorescence microscopy images before and after the treatment of PLA_2 were obtained.

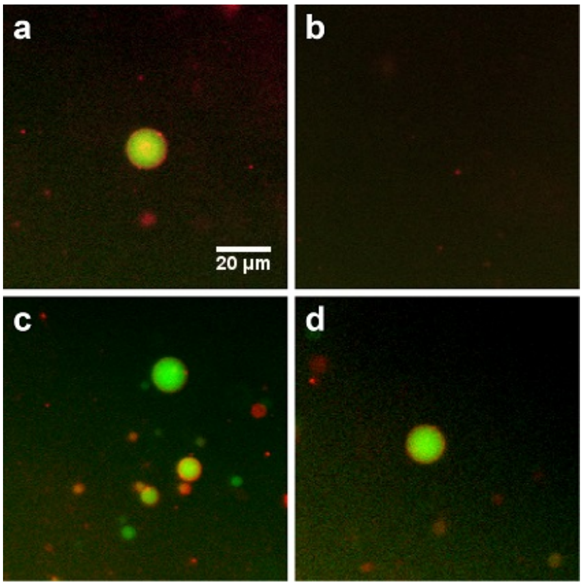

Figure S6. Dithioether PC GUVs shows resistance against PLA_2 hydrolysis, while DMPC GUVs were fully hydrolyzed after the treatment. Fluorescence microscopy images of merged DesRed and GFP channels illustrating DMPC GUVs after undergoing treatment with a) 0 μg/mL PLA_2 and b) 10 μg/mL PLA_2. Fluorescence microscopy images of merged DesRed and GFP channels illustrating C14:0 dithioether phospholipid **2** GUVs after undergoing treatment with c) 0 μg/ml PLA_2 and d) 10 μg/ml PLA_2.

8. HPLC Analysis of Dithioether PC Stability against PLA_2

2 mM lipid (DOPC or **5**) was suspended in 100 µL of 100 mM HEPES buffer (pH = 7.4) containing 10 mM $CaCl_2$ and 35 mM KCl. 10 µg/mL PLA_2 was added and the mixture was gently shaken for 15 min. The mixture was diluted with methanol and subject to HPLC analysis. ELSD and MS signals were simultaneously monitored to confirm the identity of the compound HPLC analysis was carried out on an Eclipse Plus C8 analytical column. HPLC gradient： 0 min – 1 min 50% *Phase A* in *Phase B* to 5% *Phase A* in *Phase B*, 1 min – 9 min, 5% *Phase A* in *Phase B*, 9 min – 10 min, 5% *Phase A* in *Phase B* to 50% *Phase A* in *Phase B*. (*Phase A*: H_2O with 0.1% formic acid, *Phase B*: MeOH with 0.1% formic acid).

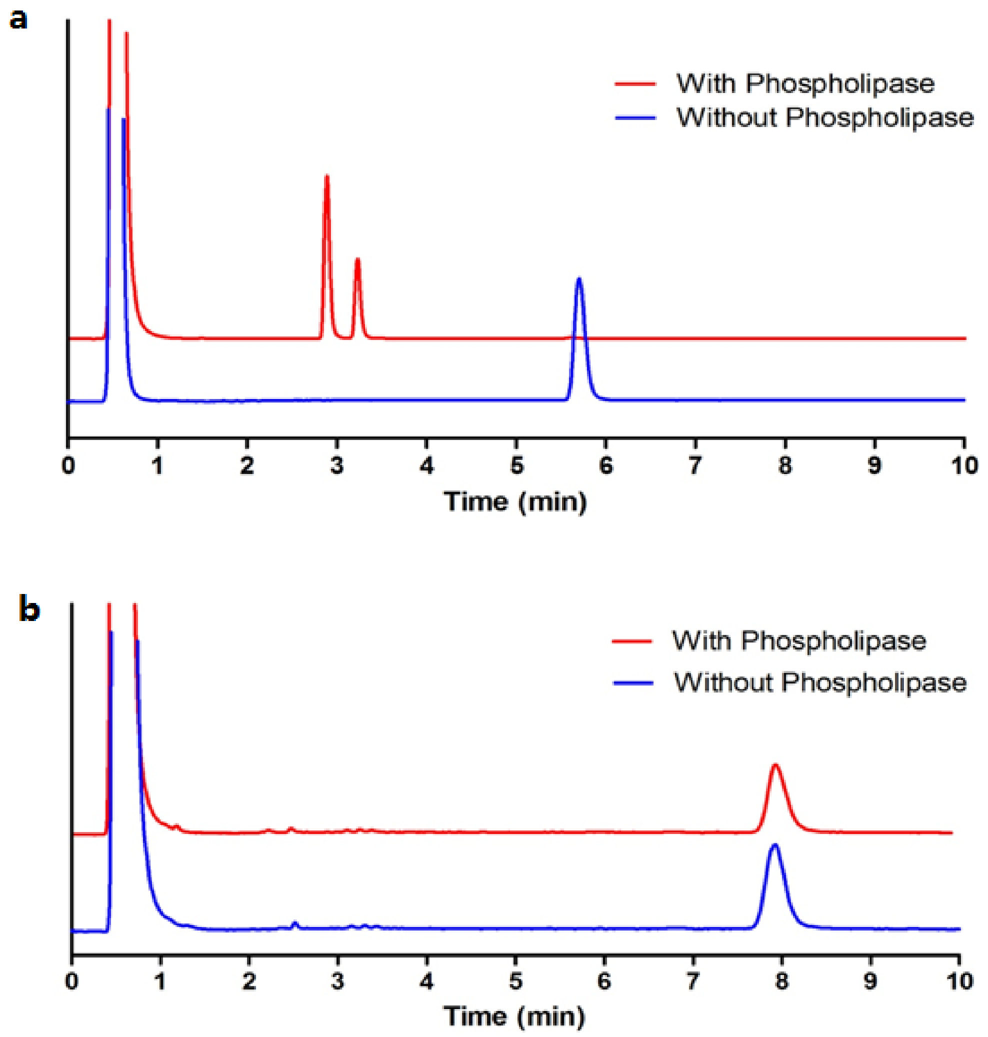

Figure S7. HPLC-ELSD signals for PLA_2 treatment of DOPC and C18:1 dithioether PC **5**. a) DOPC before (blue) and after (red) the treatment of 10 µg/ml PLA_2. Retention time for DOPC is 5.7 min. The hydrolysis of DOPC by PLA_2 afford C18:1 lyso PC (2.9 min) and oleic acid (3.2 min). b) **5** before (blue) and after (red) the treatment of 10 µg/ml PLA_2. No hydrolysis was observed. Retention time for C18:1 dithioether PC **5** is 7.9 min.

10. Live Cell Imaging

Chinese hamster ovary (CHO) cells were incubated in a 96 well tissue culture plate in cDMEM medium (10% fetal bovine serum, 1% penicillin/streptomycin) at 37 °C in a humidified atmosphere containing 5% CO_2 for 24 h to adhere to the plate. After allowing the cells to adhere, the media was aspirated. The cells were incubated in cDMEM and 5 μM Texas Red DHPE and 5 μM **8** for 1 h at 37°C in a humidified atmosphere containing 5% CO_2 protected from light. The media was aspirated, and the cells were washed twice with HBSS (Invitrogen, Carlsbad, CA) before imaging.

11. Cell Viability Study

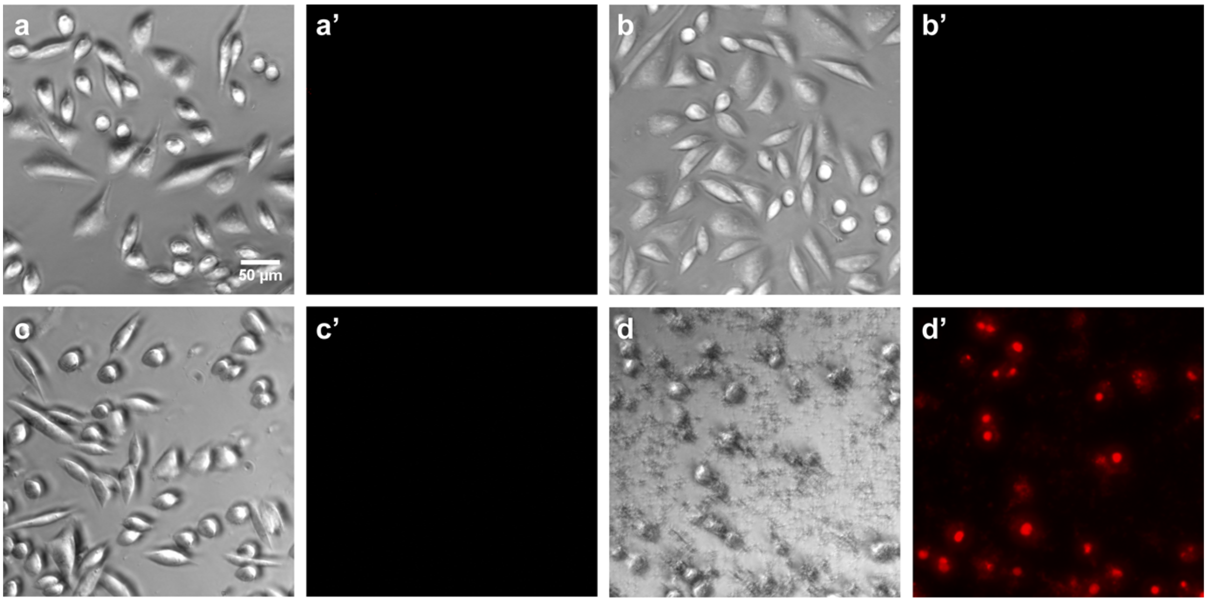

Figure S8. Membrane integrity assay by DNA staining dye propidium iodide. CHO cells were incubated with different additives for 24 h, followed by 1 h incubation with propidium iodide. The brightfield images indicated that the cells with a) no additives as control, b) 500 μg/ml POPC and c) 500 μg/ml **5** were adherent to the surface. The cells treated with d) Lipofectamine 2000 were fully ruptured. Images to the right of each brightfield image **a'-d'** are the same samples in the DesRed channel visualizing propidium iodide stain. Cells with no fluorescent stains in **a'**, **b'** and **c'** suggest that they are fully alive. Conversely, the cells in **d'** are fluorescent indicating that they are dead.

12. Supplementary Videos

12.1 Video 1. Shrinkage of a GUV encapsulated with GFP. A GUV shrinkage experiment was performed as described in the experimental section. Fluorescence microscopy imaging demonstrates that after treating the vesicles with PLA_2, the GUV begins to shrink as the DOPC is hydrolyzed and removed from membrane. GFP readily leaks out of the GUV. The real-time duration captured in the video recording is 21.3 min.

12.2 Video 2. Shrinkage of a GUV encapsulated with 100 nm red fluorescent sulfate-modified polystyrene nanospheres. A GUV shrinkage experiment was performed as described in the experimental section. Fluorescence microscopy imaging demonstrates that after treating the vesicles with PLA_2, the GUV begins to shrink as the DOPC is hydrolyzed and removed from membrane. 100 nm red fluorescent sulfate-modified polystyrene nanospheres are contained and concentrated in the GUV. The real-time duration captured in the video recording is 17 min.

13. NMR Spectra for Synthesized Material

13.1 C12:0 dithioether PC1

^{1}H NMR

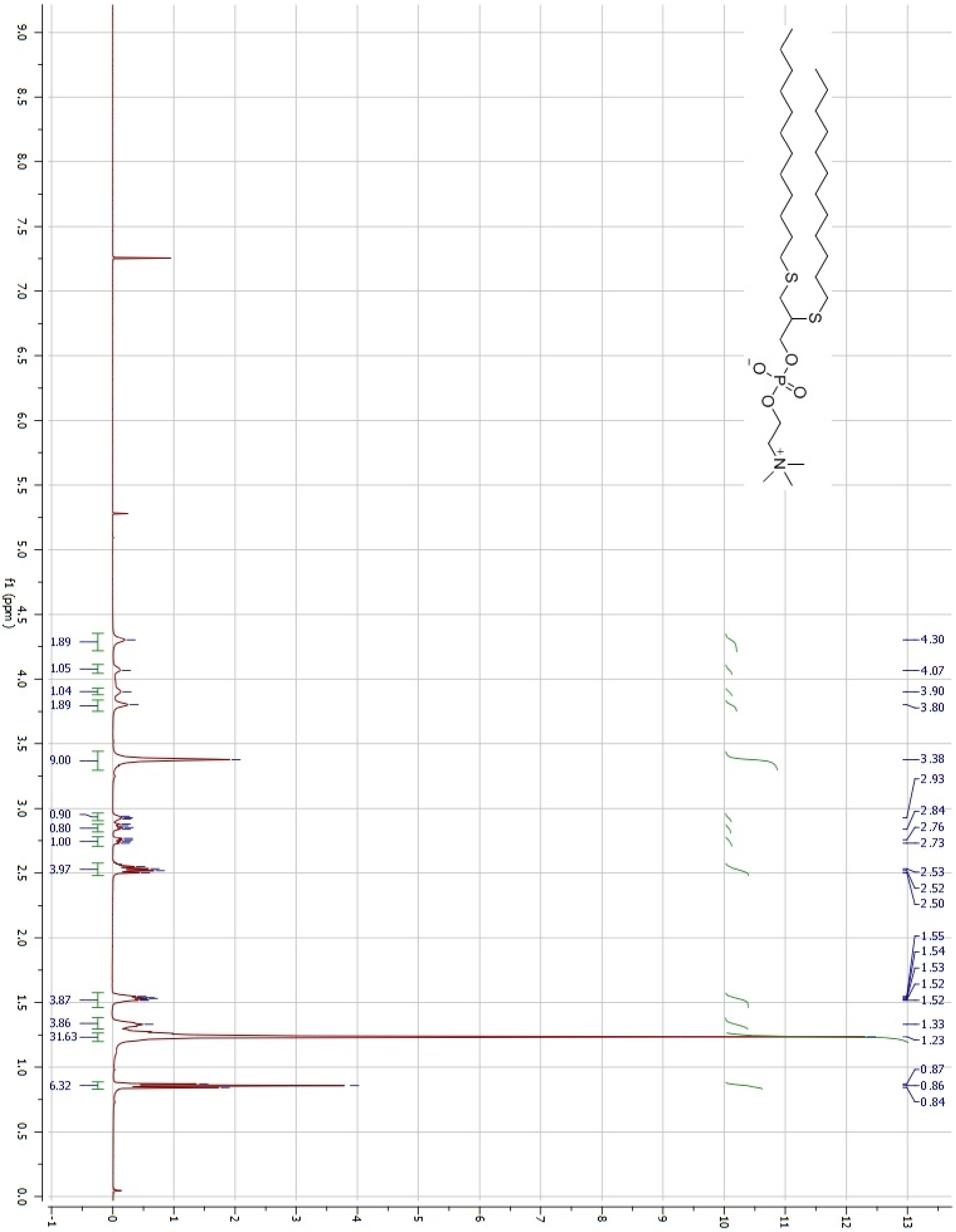

^{13}C NMR

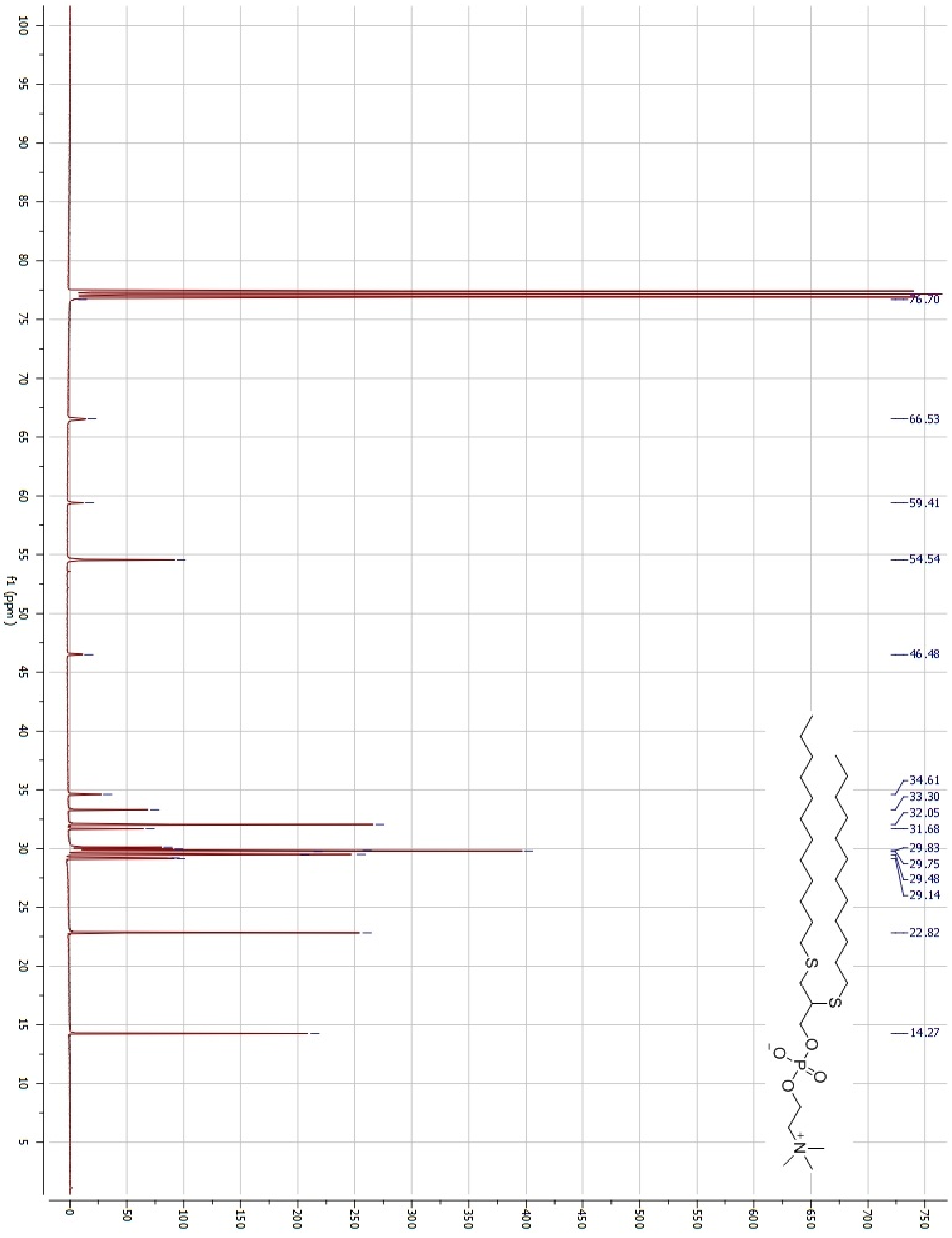
76.70
66.53
59.41
54.54
46.48
34.61
33.30
32.05
31.68
29.83
29.75
29.48
29.14
22.82
14.27
f1 (ppm)

13.2 C14:0 dithioether PC **2**

^{1}H NMR

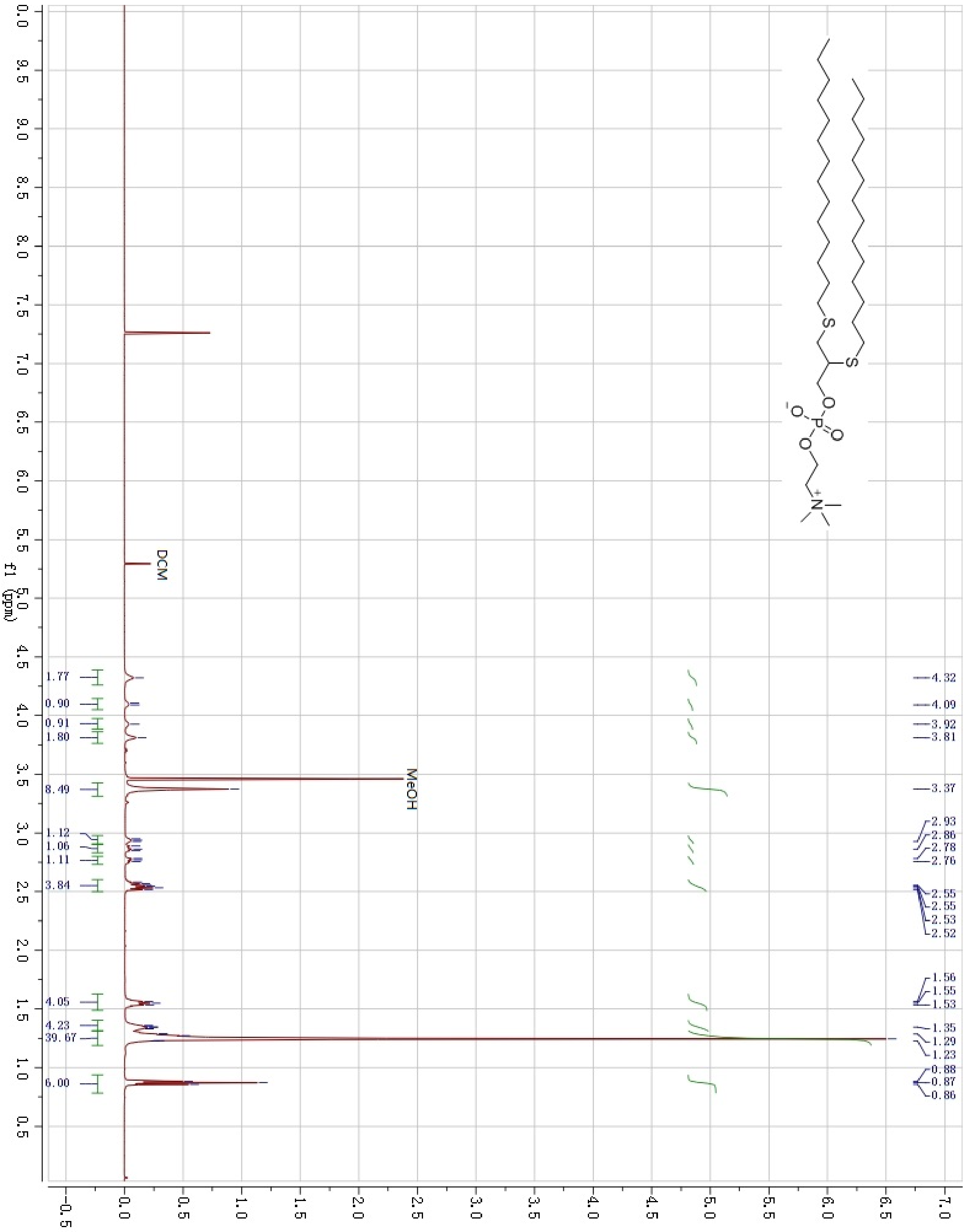

^{13}C NMR

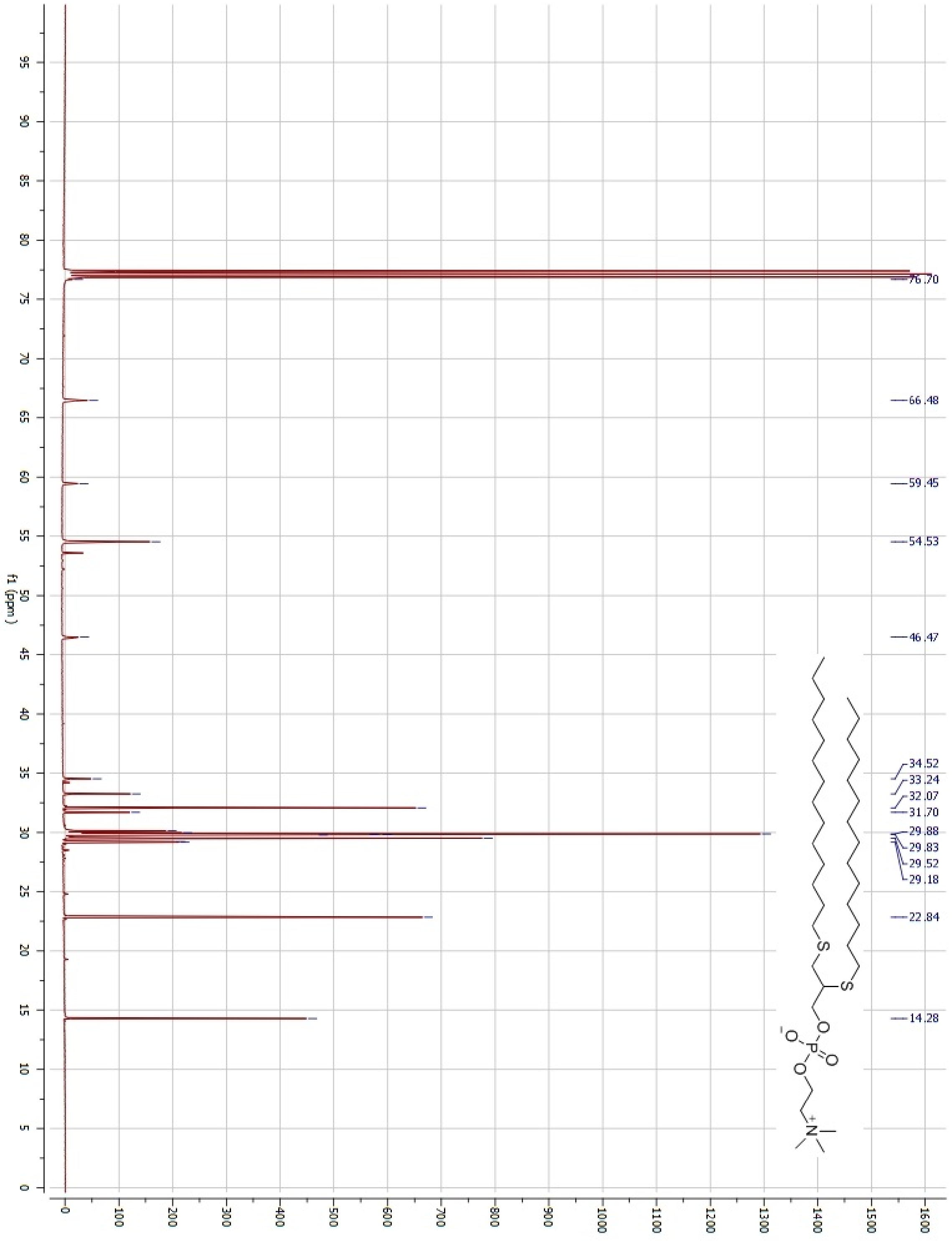
76.70
66.48
59.45
54.53
46.47
34.52
33.24
32.07
31.70
29.88
29.83
29.52
29.18
22.84
14.28
f1 (ppm)

13.3 C16:0 dithioether PC **3**

^{1}H NMR

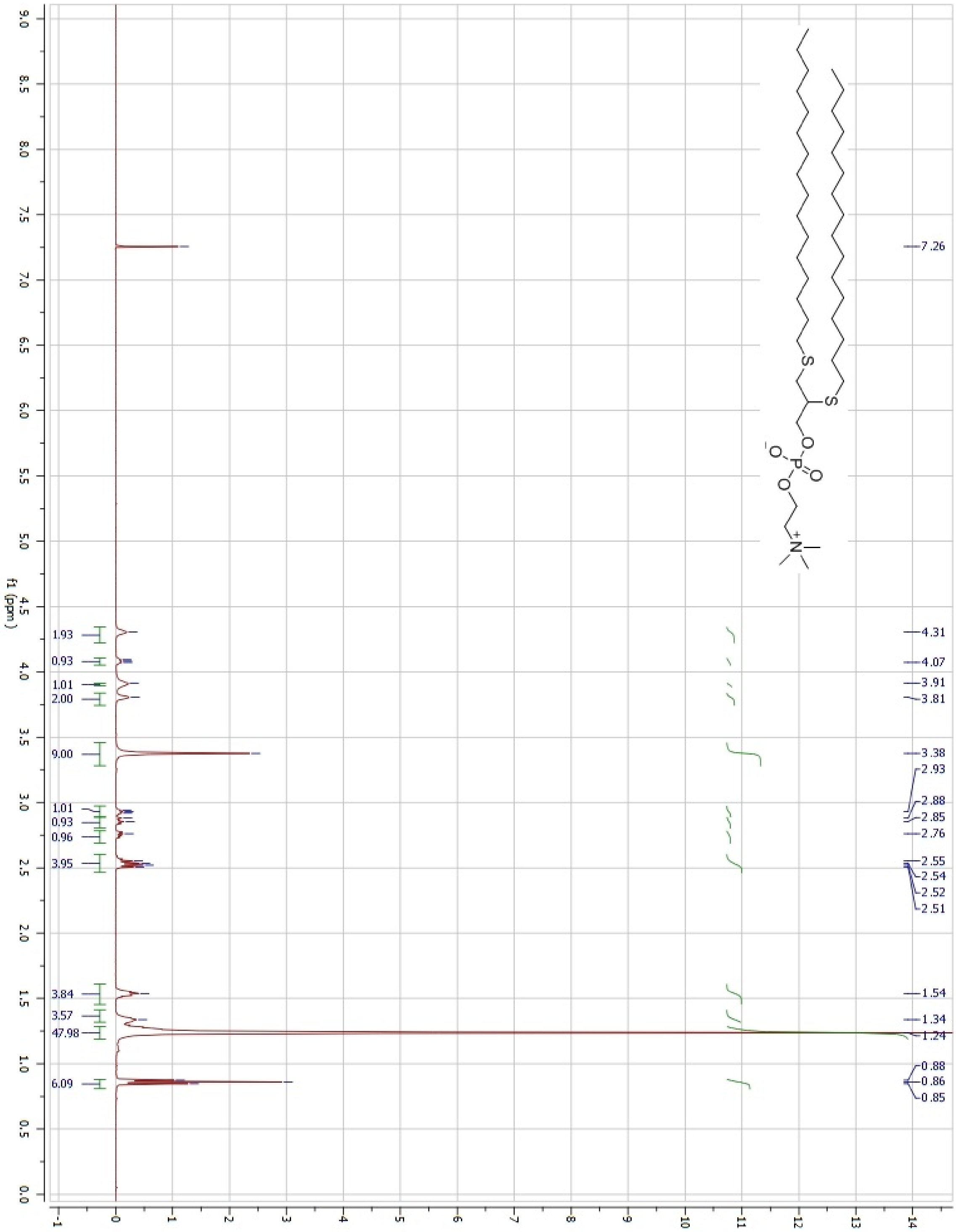

^{13}C NMR

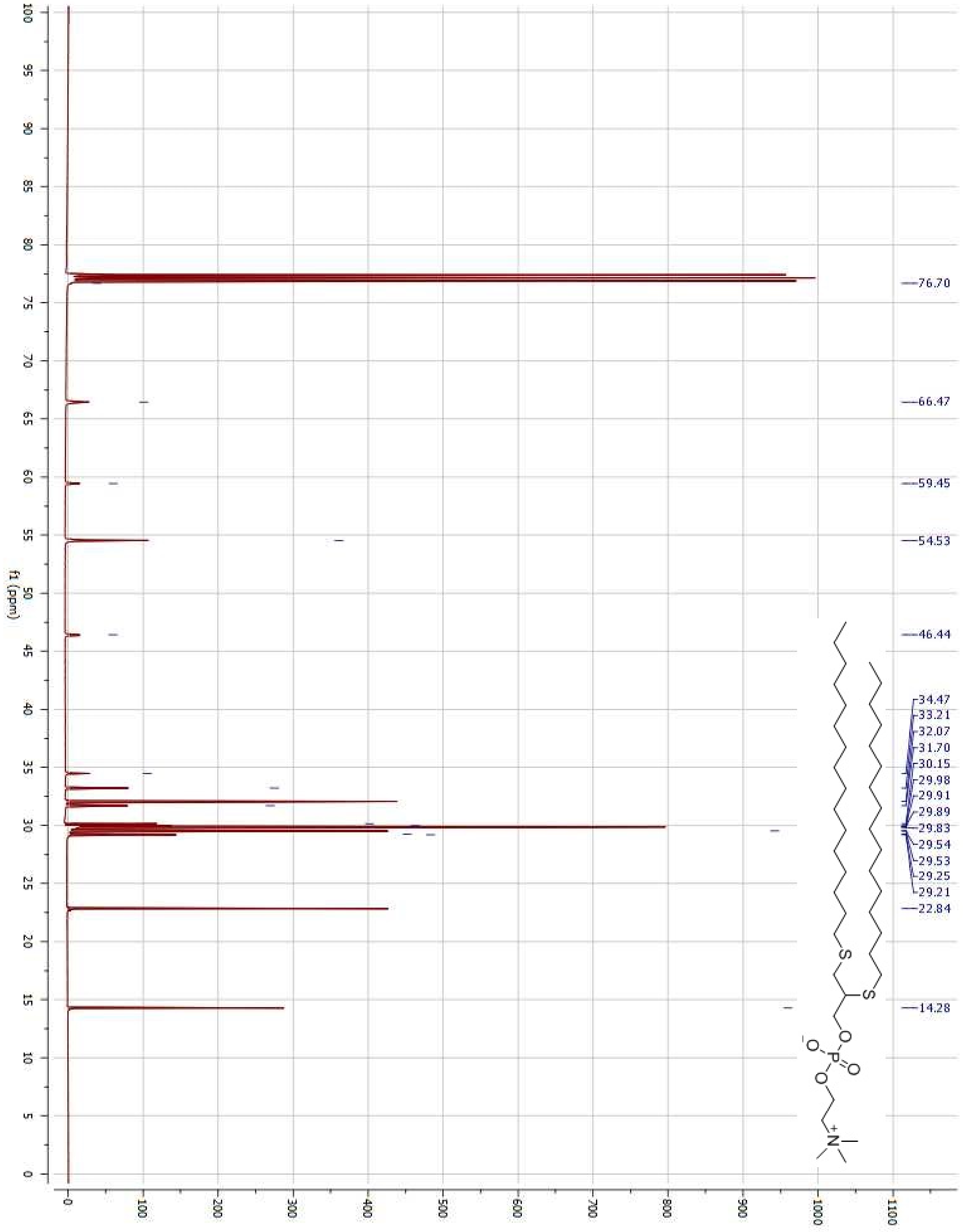

76.70
66.47
59.45
54.53
46.44
34.47
33.21
32.07
31.70
30.15
29.98
29.91
29.89
29.83
29.54
29.53
29.25
29.21
22.84
14.28
f1 (ppm)

13.4 C18:0 dithioether PC **4**

^{1}H NMR

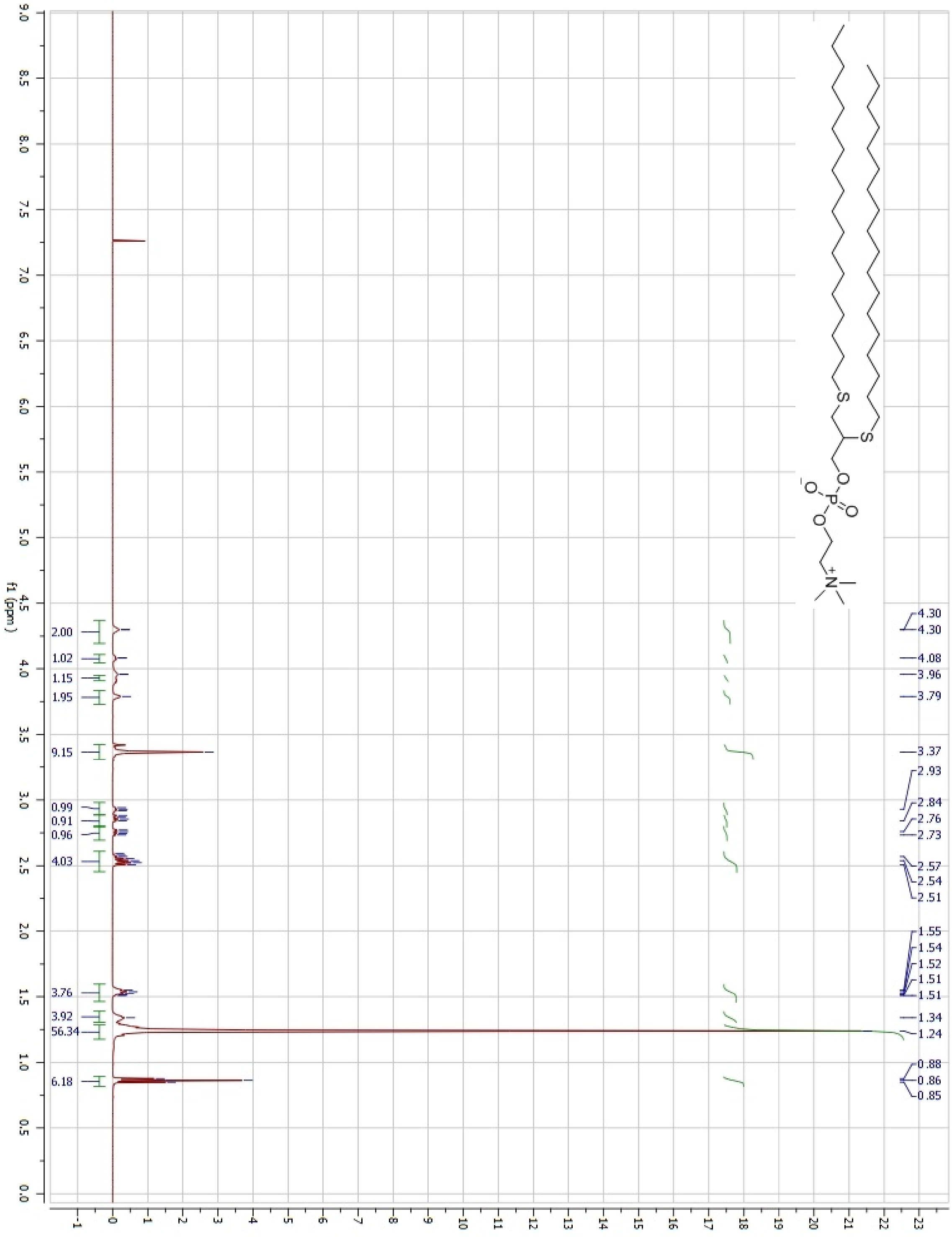

^{13}C NMR

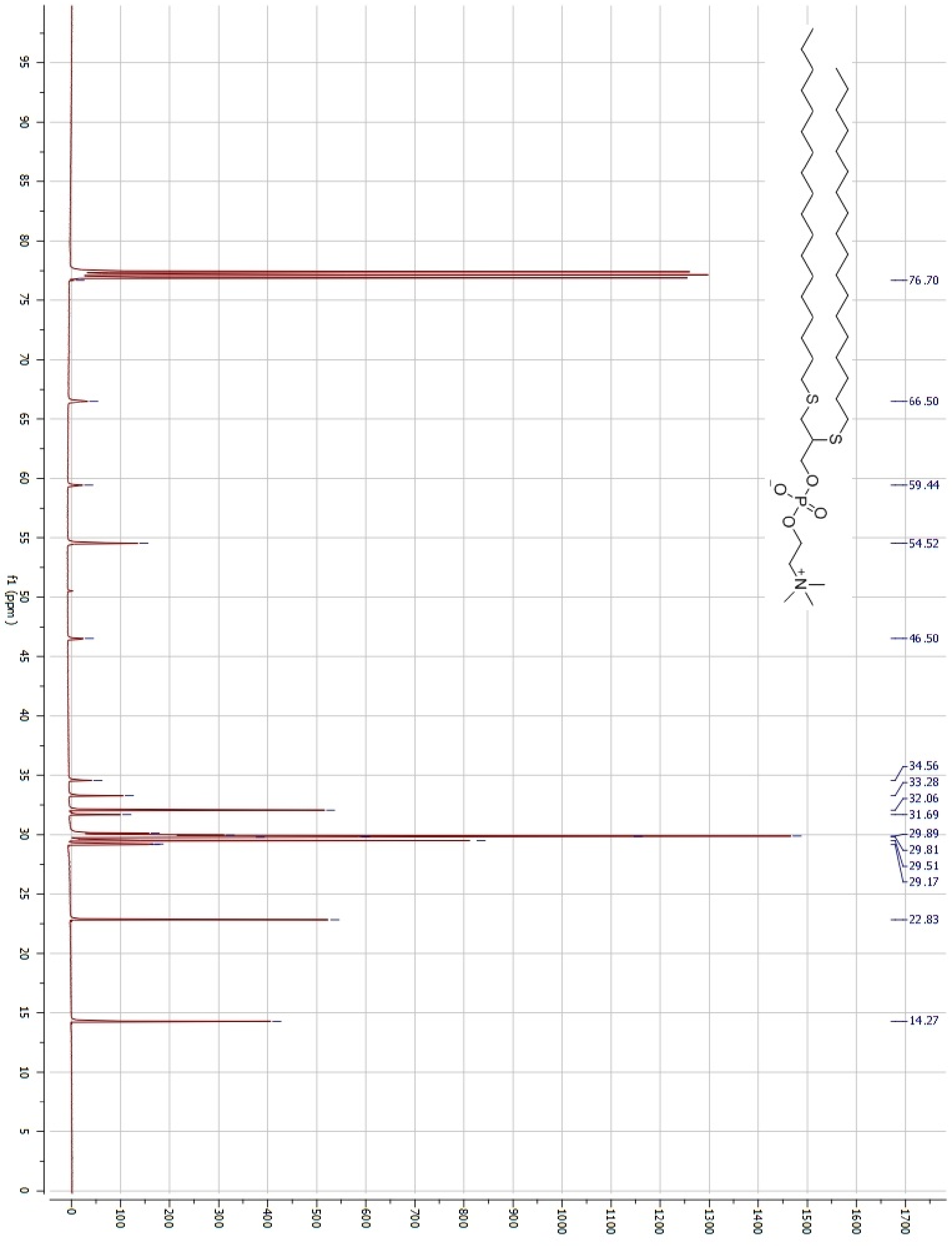
76.70
66.50
59.44
54.52
46.50
34.56
33.28
32.06
31.69
29.89
29.81
29.51
29.17
22.83
14.27
f1 (ppm)

\

13.5 C18:1 dithioether PC **5**

^{1}H NMR

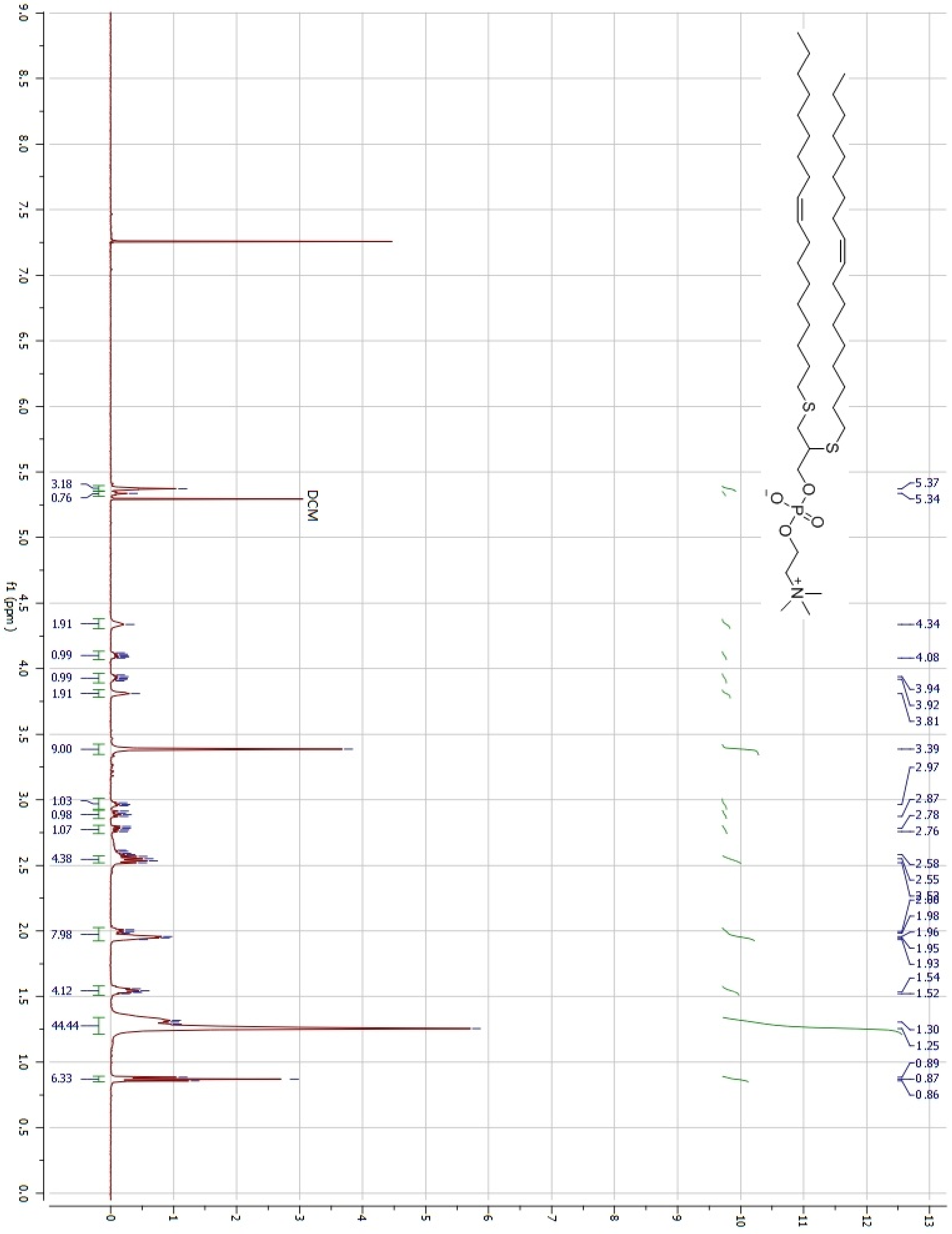

^{13}C NMR

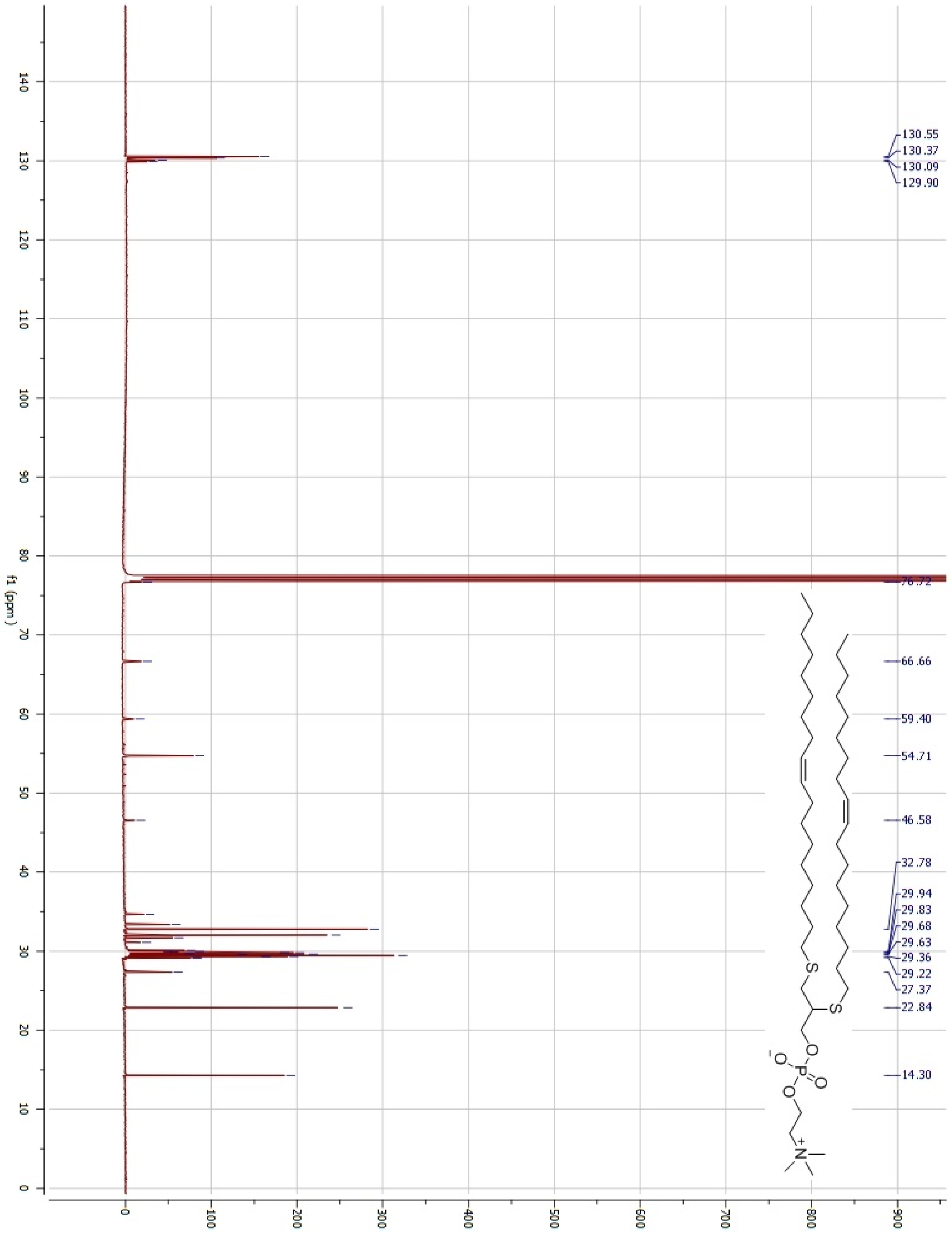

130.55
130.37
130.09
129.90
76.72
66.66
59.40
54.71
46.58
32.78
29.94
29.83
29.68
29.63
29.36
29.22
27.37
22.84
14.30
f1 (ppm)

13.6 Acetyl-Protected Glucopyranosyl Lipid **6**

^{1}H NMR

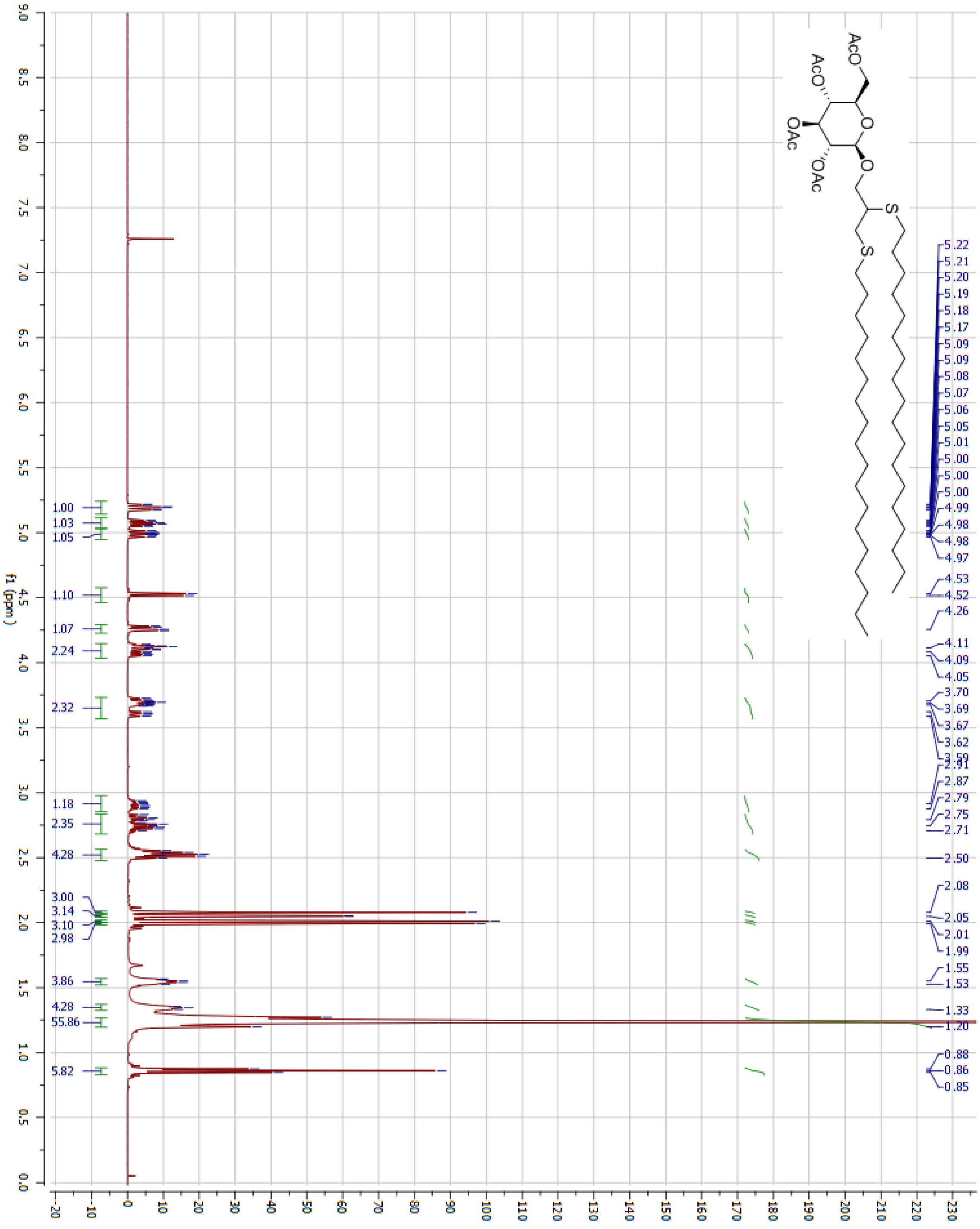

^{13}C NMR

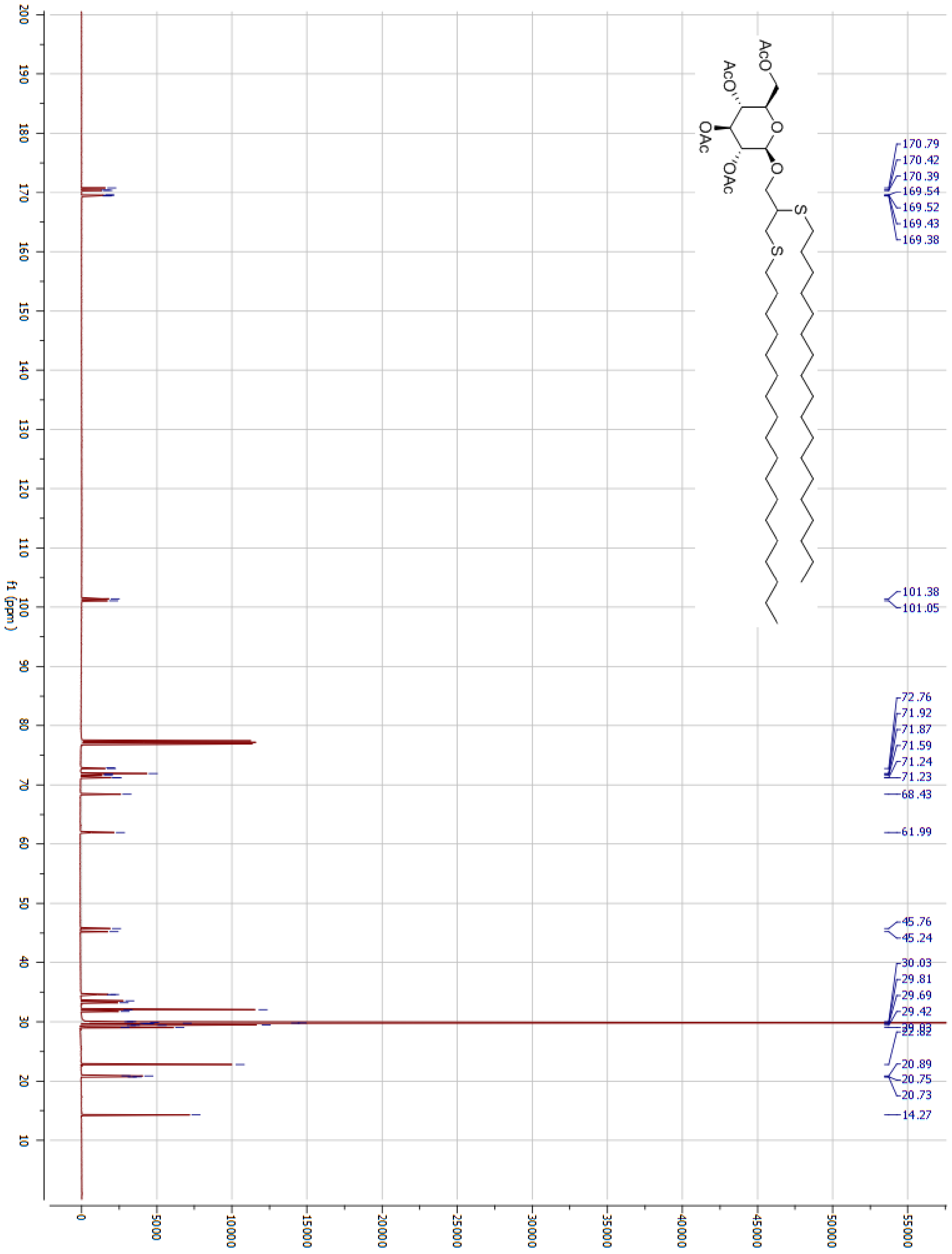
AcO
AcO
OAc
OAc
O
O
S
S
170.79
170.42
170.39
169.54
169.52
169.43
169.38
101.38
101.05
72.76
71.92
71.87
71.59
71.24
71.23
68.43
61.99
45.76
45.24
30.03
29.81
29.69
29.42
22.82
20.89
20.75
20.73
14.27
f1 (ppm)

13.7 Protection-Free Glucopyranosyl Lipid **7**

^{1}H NMR

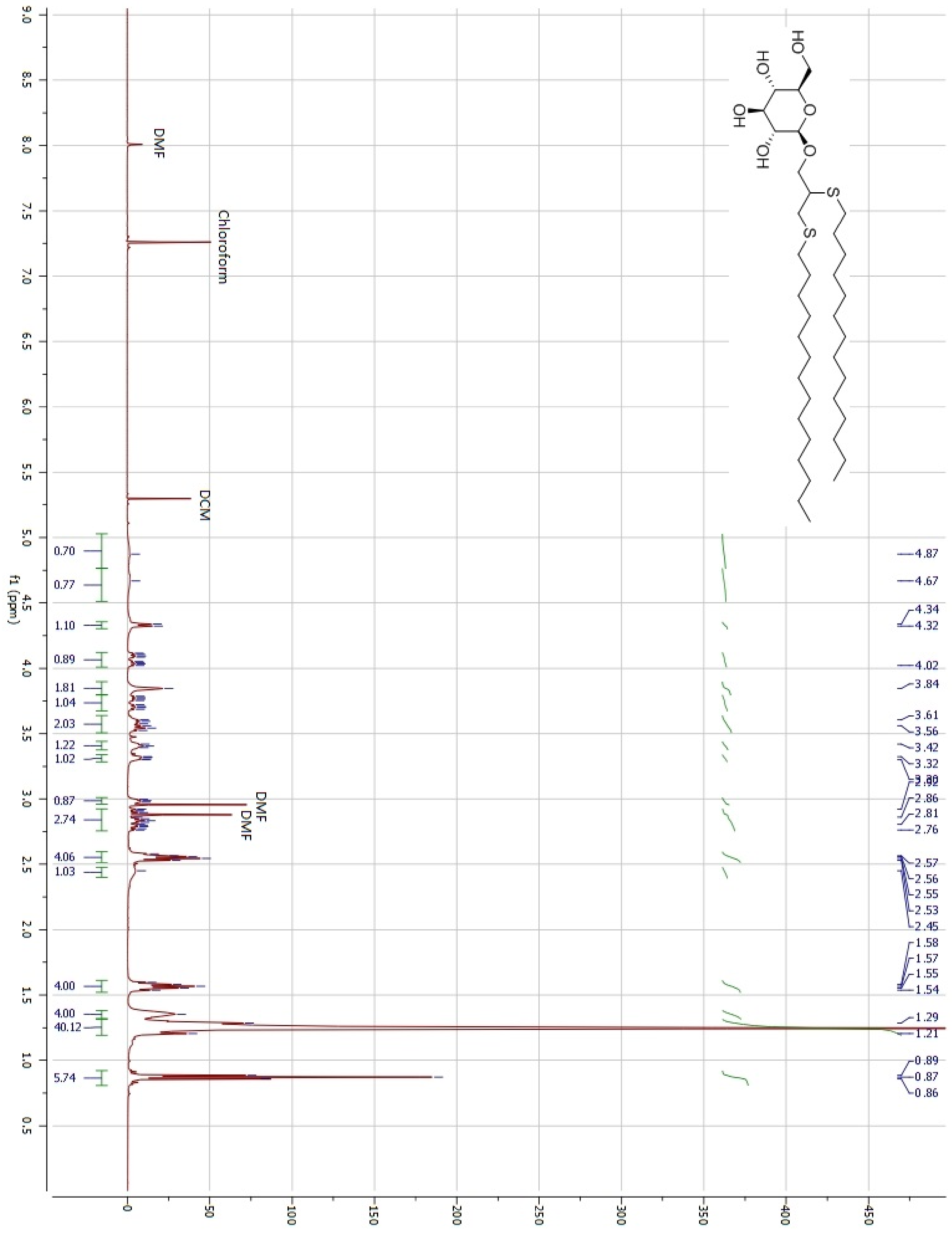

^{13}C NMR

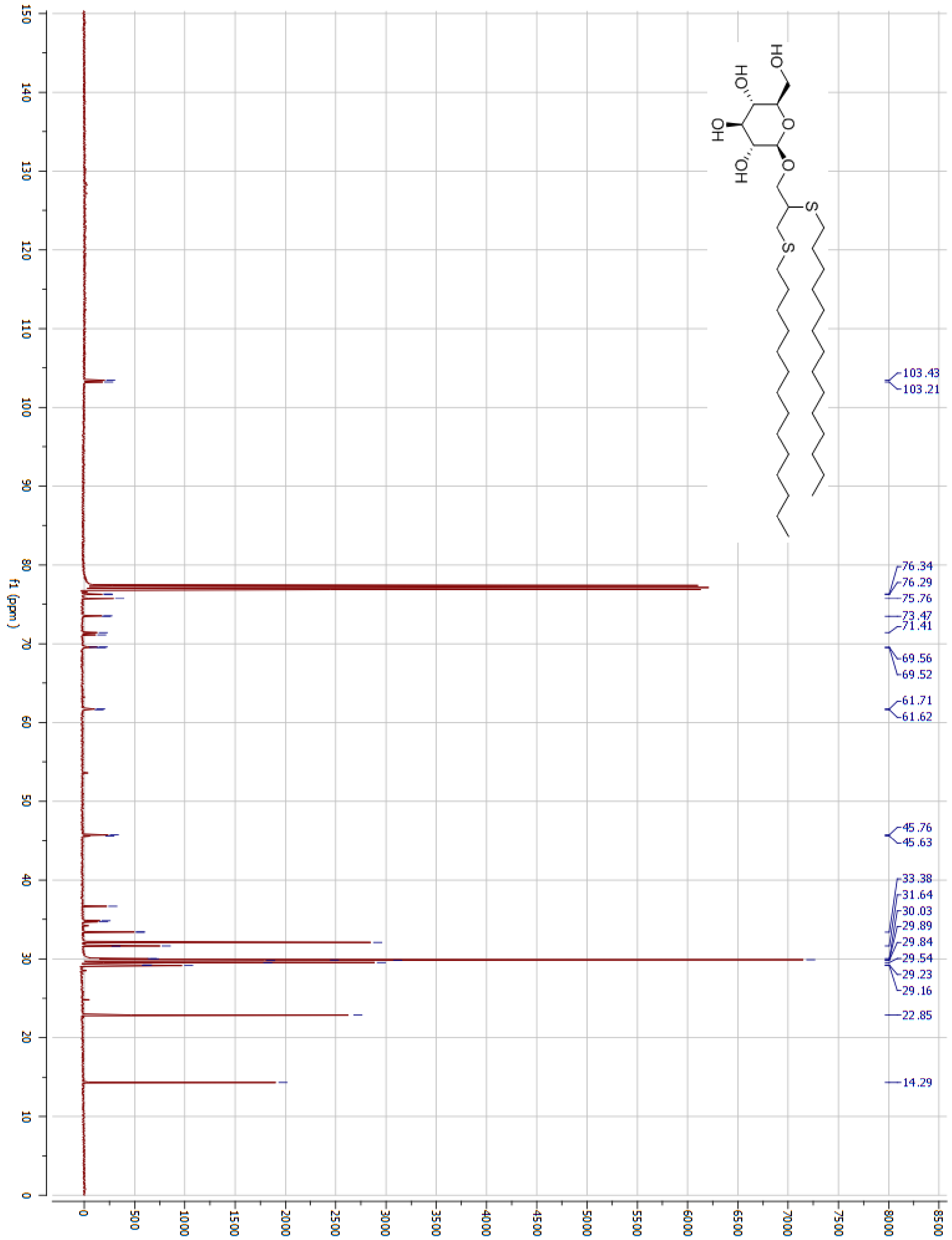
103.43
103.21
76.34
76.29
75.76
73.47
71.41
69.56
69.52
61.71
61.62
45.76
45.63
33.38
31.64
30.03
29.89
29.84
29.54
29.23
29.16
22.85
14.29
f1 (ppm)

13.8 Oregon Green C12:0 Dithioether Lipid **8**

^{1}H NMR

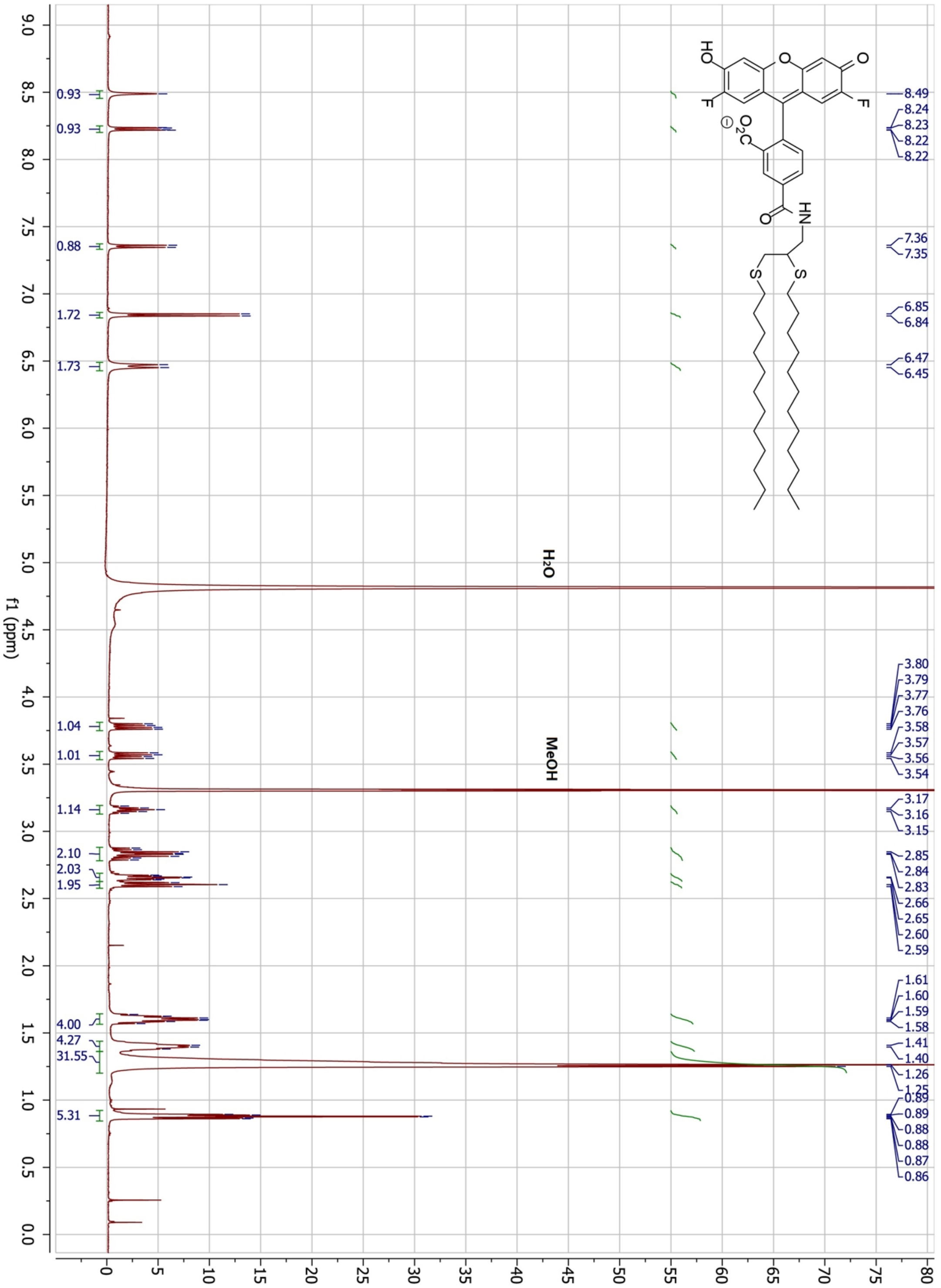

14. Supporting Information References:

(1) Yu, X.; Liu, Z.; Janzen, J.; Chafeeva, I.; Horte, S.; Chen, W.; Kainthan, R. K.; Kizhakkedathu, J. N.; Brooks, D. E. *Nat. Mater.* **2012**, *11*, 468–476.

(2) Daly, R.; Vaz, G.; Davies, A. M.; Senge, M. O.; Scanlan, E. M. *Chemistry* **2012**, *18*, 14671–14679.

(3) Naik, S. S.; Chan, J. W.; Comer, C.; Hoyle, C. E.; Savin, D. A. *Polym. Chem.* **2011**, *2*, 303.

(4) Pautot, S.; Frisken, B. J.; Weitz, D. A. **2003**, 2870–2879.

(5) Shin, J.; Noireaux, V. *J. Biol. Eng.* **2010**, *4*, 8.
